# Divvying up the pie: Tissue nutrient content is related to its parasite load

**DOI:** 10.1002/ece3.11122

**Published:** 2024-05-20

**Authors:** Adrienne Stanley, Shaley Valentine, Charlotte F. Narr

**Affiliations:** ^1^ School of Biological Sciences Southern Illinois University Carbondale IL USA; ^2^ Illinois River Biological Station Illinois Natural History Survey Havana IL USA

**Keywords:** ecological stoichiometry, infection site, *Micropterus salmoides*, parasite host interactions

## Abstract

The nutrient content of host resources can influence the abundance of parasites within an ecosystem, but linking specific nutrients in a host to the abundance of different parasite taxa remains a challenge. Here, we work to forge this link by quantifying the relationship between the nutrient content of specific infection sites and the abundance of multiple parasite taxa within the digestive tract of largemouth bass (*Micropterus salmoides*) collected from the Mississippi River. To generate a mechanistic understanding of these relationships, we tested four basic predictions: (1) the nutrient content of different host tissues (infection sites) varies within and across hosts, (2) the nutrient content of parasite genera differs from that of their host tissue(s), (3) the nutrient content of parasite genera differ from one another and (4) the nutrient content of host tissues is related to the nutrient content and abundance of parasite genera. We found support for each of these predictions. We found stoichiometric differences between the digestive tissues we examined. We also found that across hosts, intestine and pyloric caeca C:N ratios increased and %N decreased with fish condition factor. Both of the actively feeding parasitic genera we measured had lower C:N ratios compared to both their host tissue and other encysted/non‐reproductive genera, suggesting the potential for N limitation of these parasites in the intestines or pyloric caeca of hosts. Consistent with this possibility, we found that the total number of actively feeding parasitic worms in the pyloric caeca increased with that tissue's N:P ratio (but was not related to host condition factor). Our results suggest that parasites encounter significant variation in nutrient content within and across hosts and that this variation may influence the abundance of actively feeding parasites. This work highlights the need for additional empirical comparisons of parasite stoichiometry across tissues and individual hosts.

## Graphical abstract

We investigated how the size and composition of parasitic infracommunities are affected by the nutrient content of their infection sites. We expected infracommunities to be dominated by parasites that were stoichiometrically similar to their infection sites. However, we found that parasites, themselves, can vary in nutrient content and that the size of entire infracommunities in a single organ can respond to variation in that organ's N:P ratio.

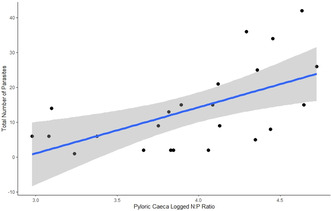

## 1 INTRODUCTION

Determining the consequences of aquatic nutrient loading on parasitic organisms has implications for both public and ecosystem health (Dobson et al., 2008). There is a clear link between rising nutrient levels and infectious disease (Altman & Byers, 2014; Johnson et al., 2010), but the mechanisms behind this link are murky (Johnson et al., 2010; McKenzie & Townsend, 2007). Here, we work to clarify this relationship by examining how variation in the nutrient content of host tissues is related to the nutrient content of parasite taxa and size of parasitic infracommunities and in each tissue.

The framework of ecological stoichiometry can be used to help predict the effects of shifting resources on host–parasite interactions (Aalto et al., 2015; Bernot & Poulin, 2018; Frenken et al., 2021; Sanders & Taylor, 2018). In general, this framework uses the elemental composition (primarily the contents and ratios of carbon (C), nitrogen (N) and phosphorus (P)) of interacting species to predict the outcome of interactions like predation and competition on species success (Sterner & Elser, 2002). We can extend this framework to parasites by comparing the elemental composition of parasitic organisms to the tissues they live in. When consuming the tissues they inhabit, stoichiometric theory predicts that parasites will replicate faster in tissues similar to their own stoichiometric composition (Aalto et al., 2015). As a result, variation in host nutrient content may alter the replication rate of the parasite (Frost et al., 2008). Whether a parasitic organism consumes host tissue directly or instead feeds on the circulatory or digestive fluids around it is often unclear (Von Brand, 1952). However, we anticipate that variation in the stoichiometric composition of tissues across hosts is reflective of the variation in nutrients made available to these tissues from the same circulatory or digestive tissues that parasitic organisms would feed on. If so, and if parasites are not strictly stoichiometrically homeostatic, then it is possible that parasite and host stoichiometry might covary across hosts. Unfortunately, we lack empirical data on the elemental composition of most parasites and the tissues they inhabit so the application of stoichiometric theory to parasites remains underdeveloped, and there is very little work comparing host and parasite elemental composition (Frenken et al., 2021). Here, we work to advance the application of stoichiometric theory to host–parasite systems by quantifying variation in the elemental composition of specific host tissues across and within hosts and comparing this variation to that of the parasitic organisms that occupy these tissues. Then, we assess whether these trends can help predict parasite abundance.

Characteristics of individual hosts and the role of specific host tissues likely contribute to variation in the elemental composition of infection sites. In animals, characteristics like size and condition are expected to influence body N:P ratios (Elser et al., 1996a, 1996b). Because these characteristics are, in turn, likely shaped by an organism's diet and or environment (Raubenheimer & Simpson, 2004), shifts in a host's environment may influence a parasite's access to resources via, for example, shifts in host stoichiometry or growth rate (Smith et al., 2015). However, the function of specific animal tissues dictates their stoichiometry (Færøvig & Hessen, 2003), and we suspect that it also dictates the amount of stoichiometric variation within the same tissue across hosts. For example, we suspect that the stoichiometry of tissues that function in nutrient absorption will be more closely tied to variation in host characteristics (e.g. host condition) than the stoichiometry of tissues that do not function directly in nutrient absorption. If so, a host's nutritional environment should exert more pressure on the parasitic infracommunities in these more variable tissues, and we would expect to see clearer relationships between tissue stoichiometry and the elemental composition and number of parasites occupying these tissues than those occupying other less variable tissues.

Characteristics of parasites also contribute to variation in the elemental composition of their bodies (Bernot & Poulin, 2018; Paseka & Grunberg, 2019). A parasite's elemental composition may reflect the metabolic needs of that parasite which, in turn, are affected by its life stage (Zuzarte‐Luís & Mota, 2018). For example, in macroparasites, larval stages primarily invest in growth while adult stages invest in reproduction (Poulin, 2011). Because growth and reproduction require different suites of biomolecules, different life stages possess different elemental compositions (Paseka & Grunberg, 2019). The elemental composition of a parasite may also reflect shifts in the nutrients available to it within a host. Just as some free‐living species are more capable of modifying their nutrient stores (i.e. are less stoichiometrically homeostatic) than others (Persson et al., 2010), it is possible that the strength of stoichiometric homeostasis varies across parasite taxa and life stages. For example, after an initial growth phase, encysted parasites likely feed only minimally off their hosts (Lowenberger & Rau, 1993). This metabolic independence should enable encysted parasites to maintain stronger stoichiometric homeostasis than actively feeding parasites. To our knowledge, the only study that compared the stoichiometric composition of a parasite to the tissues of its host after modifying the hosts' diet suggested that the stoichiometry of actively feeding trematodes is flexible (Narr & Krist, 2015). This study suggested that a trematode parasite may store more of a critical nutrient when it is abundant and there is less competition for this resource from the host. Thus, there is a clear need to contextualize variation in parasite elemental composition within the nutritional environment of the host by quantifying the mismatch between parasite and host stoichiometry.

In this study, we investigated the relationship between the stoichiometry of multiple largemouth bass (*Micropterus salmoides*) digestive tissues and the parasitic infracommunities that infect them. Largemouth bass exhibited substantial variation in condition because they were collected from multiple sites in the Upper Mississippi River, which has a long, well‐documented history of eutrophication (Justić et al., 2002). The alimentary canals of largemouth bass are host to a diverse group of parasites that infect multiple tissues, exist at different life stages and occupy multiple feeding groups (Costa et al., 2018; Howick & OˈBrien, 1983). We focused on the tissues of the digestive system because they are involved, to varying degrees, in food absorption and, as a result, their stoichiometry should be more tightly related to host condition than other tissues. Food absorption occurs primarily in the pyloric caeca, and to a lesser extent the intestines and stomach (Sarbahi, 1951). For comparison, we included the liver, which is not directly involved in food absorption, so we expected it to be more homeostatic than the other organs. We measured the nutrient mismatch between parasites and host tissue and compared the nutrient content of parasite genera to one another. Finally, we tested to see if the nutrient content in the host tissue was related to the nutrient content and abundance of parasitic worms or if variation in any of these variables were related to host condition.

## 2 METHODS

### 2.1 Fish collection

Largemouth bass were collected by long‐term resource monitoring element personnel as part of the Upper Mississippi River Restoration Program. Collection dates for the fish ranged from 6/20/2019 to 10/28/2019 and included young of year as well as adult fish. Fish were collected from multiple sites within three navigation pools of the Upper Mississippi River: Pool 4 (Lake City, Minnesota, RKM 1210–1283), Pool 8 (La Crosse, Wisconsin, RKM 1092–1131) and Pool 13 (Bellevue, Iowa, RKM 841–896). Personnel from the Minnesota, Wisconsin and Iowa Department of Natural Resources used standard protocols (Ratcliff et al., 2014) for fish collection. At time of collection, each fish was weighed, measured, and given a unique identifier. Fish were frozen at −20°C, thawed for gut contents analysis for a different study, and then refrozen at −20°C until we dissected them.

### 2.2 Host–parasite system

The alimentary canal of largemouth bass can be infected by several genera of parasites with differing target tissues, life stages and trophic levels. In North America, acanthocephalans like *Neoechinorhynchus* spp. and *Leptorhynchoides* spp. feed through absorption of the host's undigested food and are primarily found in the pyloric caeca or intestines (Mehlhorn, 2001). *Neoechinorhynchus* spp. use ostracods as first intermediate hosts then smaller fish, like bluegill (*Lepomis macrochirus*) as a second intermediate host (Ward, 1940). The life cycle of *Leptorhynchoides* spp. first involves amphipods but they can also encyst in fish (Van Cleave, 1920) before reaching a sexually reproductive stage in higher trophic level fish like largemouth bass. Trematodes like *Crepidostomum* spp. can be found in active feeding/reproductive stages in the stomach, pyloric caeca or intestines of largemouth bass (Klein et al., 1969) where they feed on host tissues. *Crepidostomum* spp. are known for complex multi‐host life cycles involving fingerling clams, burrowing mayflies and then fish (Choquette, 1954). Smaller fish can act as paratenic hosts for *Crepidostomum* spp. The encysted larva of the trematode *Posthodiplostomum* spp. are found in the livers of largemouth bass (Hoffman, 1958), their first intermediate host are snails and they reach a sexual reproductive stage in piscivorous birds (Miller, 1953). Nematodes like *Contracaecum* spp. are found as encysted larva that occur in the connective viscera lining the outside of the stomach and pyloric caeca of largemouth bass (Bangham, 1939), they infect fish through ingestion of infected copepods and reach sexually reproductive stages in a wide range of birds or mammals (Huizinga, 1967).

### 2.3 Fish dissection and parasite collection

Largemouth bass tissues and organs were thawed and dissected per methods described by Hoffman and Williams (1999). We separated the organs of the alimentary canal and liver from one another and then removed parasites from the organs and tissues. The stomach, intestine and pyloric caeca were scraped and then washed, and the diluent was allowed to settle in a conical container. The fluid was then decanted by pouring out the upper two‐thirds of the solution. If the remaining diluent was murky, more distilled water was added and the contents were allowed to resettle. This process was repeated until the diluent was clear, and the remaining matter was searched for parasites. Once washed, organs were viewed under a dissecting scope and searched visually for parasites. Liver tissue was pressed between two pieces of glass and examined under a dissecting scope at up to 4x magnification. When parasites were found, they were removed from the tissues of the liver, dried to a constant weight and then stored in a desiccator. Finally, the livers were also rinsed using the procedure specified above for the digestive organs. One of the parasite genera encountered, *Contracaecum* spp. occurred in the connective viscera lining the outside of the stomach and pyloric caeca. To associate *Contracaecum* spp. with the nutrient content of its host ‘tissue’, we averaged the nutrient values of the stomach and pyloric caeca. We refer to this average as ‘viscera; mesentery’ throughout.

### 2.4 Nutrient analyses

Parasites in each organ were counted, removed from the surrounding tissue, and then visually identified into genus, using Hoffman and Williams (1999). When individual parasite biomass was too small to be detected by our nutrient analyses (if the biomass of the parasite was <0.5 mg of dry weight), individual parasites were grouped with others from the same genus, fish and tissue. For eight of our samples, this still did not result in our target biomass, so parasites from the same organ in multiple hosts were combined. To incorporate data from these samples into our statistical analyses comparing organ and parasite stoichiometry, organs associated with grouped parasites were averaged and assigned a new unique fish ID.

After parasites were removed, organs were separated from the surrounding tissues, and large fat deposits were removed. All samples used for nutrient analyses (organs, tissues and parasites) were dried at 50°C to a constant weight similar to methods used by Hendrixson et al. (2007). Organ and tissue samples were ground into a homogenized powder and then subsampled for C, N and P analysis. Phosphorus analysis was conducted on a Thermo Scientific Genesys 40 Visible Spectrophotometer using the ascorbic acid molybdenum blue method following persulfate digestion (APHA, 1992), and apple leaf standards (NIST 1515) were used as a reference. Carbon and nitrogen analyses were conducted at Southern Illinois University's Core Facility for Ecological Analyses on a Thermo Scientific Flash 2000 Elemental Analyzer.

### 2.5 Statistics

Parasites found in <30% of our fish were excluded from our analyses to avoid making inference from datasets with <10 data points. All molar nutrient ratios were log transformed to improve normality and reduce the effect of outliers. We tested our prediction that the nutrient content of different host tissues and parasite genera vary in their nutrient content using separate linear mixed models that predicted each stoichiometric parameter (i.e. %C, %N and %P and C:N, C:P and N:P molar ratios) across (1) organ and (2) parasite tissues. All linear mixed models were conducted in R using the ‘lme4’ package and marginal (*R* ^2^m) and conditional *R* ^2^ (*R* ^2^c) were calculated using the ‘MuMIn’ package (Nakagawa & Schielzeth, 2013). We included the pool where the fish was collected as a random effect in each model. Because multiple organs and parasites came from the same fish, we included fish ID as a random effect as well. If models indicated a significant (*p* < .05) effect of organ or parasite genera on any stoichiometric parameter, we used the function ‘emmeans’ with Tukey corrections from the package ‘emmeans’ (Lenth, 2022) in R to identify pairwise differences between that stoichiometric parameter across organs or parasites. This function enabled us to account for the unequal numbers of fish from the separate collection pools and the effect of our random predictors in these pairwise comparisons. To investigate if fish condition factor (Fulton's Condition Factor from Ricker, 1975) accounted for any variation in the nutrient composition of fish tissues we used linear mixed models predicting the stoichiometric parameter of the tissue with pool as a random effect in each model.

To determine if the stoichiometric parameters of parasite genera differed from those of their host tissues, we calculated the ‘mismatch’ between parasite and host tissues by subtracting the stoichiometric parameter of the host organ from that of each parasite. We used linear mixed effects models to predict the stoichiometric mismatch between parasite and host based on the parasite taxa with the pool the host came from and fish ID entered as random effects into the model. We concluded that there was a difference between parasite and host tissues for a stoichiometric parameter when these confidence intervals did not overlap with zero.

We tested our prediction that host and parasite nutrient content were correlated using linear mixed models (with fish ID held as a random effect) that predicted each stoichiometric parameter of the parasite as a function of variation in the same parameter in the organ that parasite inhabited at the time of dissection. For parasites that were found in multiple different types of organs (i.e. parasites found in the intestines and stomach), we used linear mixed models to determine if organ type was related to the nutrient content of the parasite and, again, included fish id as a random effect. If we detected a significant effect of organ type on the parameters of parasites that inhabited multiple organs within a host, we conducted emmeans pairwise analysis with Tukey corrections to determine differences in these parameters between organs.

We tested our prediction that parasite loads vary in response to host organ stoichiometry, host condition or the stoichiometric mismatches between parasite and host using linear mixed effect models. Because we expected the process that affected parasite load to differ from the process that affected the presence of parasites within a fish, fish without the target parasite were excluded from all parasite load analyses. To determine if variation in the load of each parasite was well‐explained by any of the stoichiometric parameters we measured in organs, we created a set of eight candidate linear regression models predicting the load of each parasite genera within an organ and compared them using Akaike's Information Criteria corrected for small sample sizes (AICc). To avoid overfitting and multicollinearity, each model included one of the 6 stoichiometric parameters (%C, %N, %P, C:N, C:P and N:P) of the organ and collection pool as a random effect. For comparison, we also added a model with just collection site and a null model (including no predictors) to our candidate set. We used the same set of models and model selection process to assess the effect of organ stoichiometric parameters on the size of the total parasite infracommunity within each organ. We also used a linear mixed effect model with pool as a random effect to determine if host condition factor influenced the size of the parasitic infracommunity in each organ. To determine if the mismatch between organ and parasite stoichiometry helped explain parasite load we again created a set of eight linear models including fish length and collection pool, but we replaced the stoichiometric parameter of the organs with the calculated mismatch in each model. These models were also compared to a model including just collection site and a null model. For all candidate sets, models with delta AICc values <2 were considered top models. Data were analysed with R version 4.2.0.

## 3 RESULTS

### 3.1 Organ and parasite stoichiometry

**FIGURE 1:**
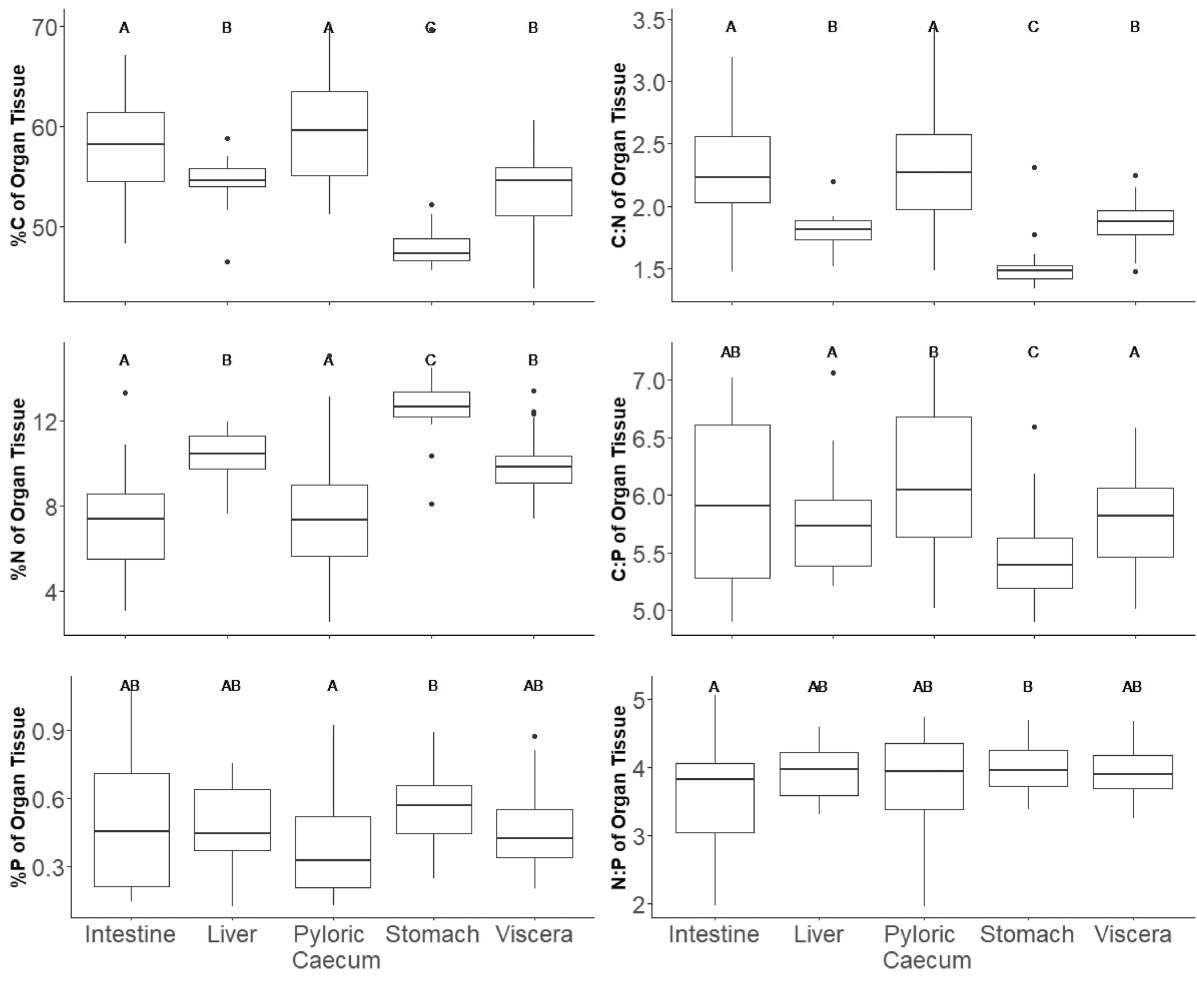
Carbon, nitrogen and phosphorus content and their ratios compared across organs from 30 largemouth bass collected from the Upper Mississippi River. Emmeans with Tukey corrections was used to determine differences; different letters denote significant differences.

We found differences in the %C, %N, %P, C:N, C:P and N:P of the largemouth bass organs included in our study (%C *F*_(4,120.67)_ = 43.72, *p* < .001, *R* ^2^m = .49, *R* ^2^c = .58; %N *F*_(4,119.37)_ = 52.44, *p* ≤ .001, *R* ^2^m = .51, *R* ^2^c = .64; %P *F*_(4,118.19)_ = 5.86, *p* ≤ .001, *R* ^2^m = .08, *R* ^2^c = .48; C:N *F*_(4,120.05)_ = 44.025 *p* < .001, *R* ^2^m = .48, *R* ^2^c = .59; C:P *F*_(4,118.28)_ = 14.476 *p* < .001, *R* ^2^m = .18, *R* ^2^c = .55; N:P *F*_(4,118.5)_ = 2.92 *p* = .02, *R* ^2^m = .04, *R* ^2^c = .44, Figure 1, Table A1). Specifically, largemouth bass intestines and pyloric caeca had higher %C and C:N ratios than their livers and stomachs. The stomachs of largemouth bass had the lowest %C of any of the tissues we analysed. Conversely, stomachs and then livers had the highest %N and stomachs had the lowest C:P ratio among the tissues we analysed.

**FIGURE 2:**
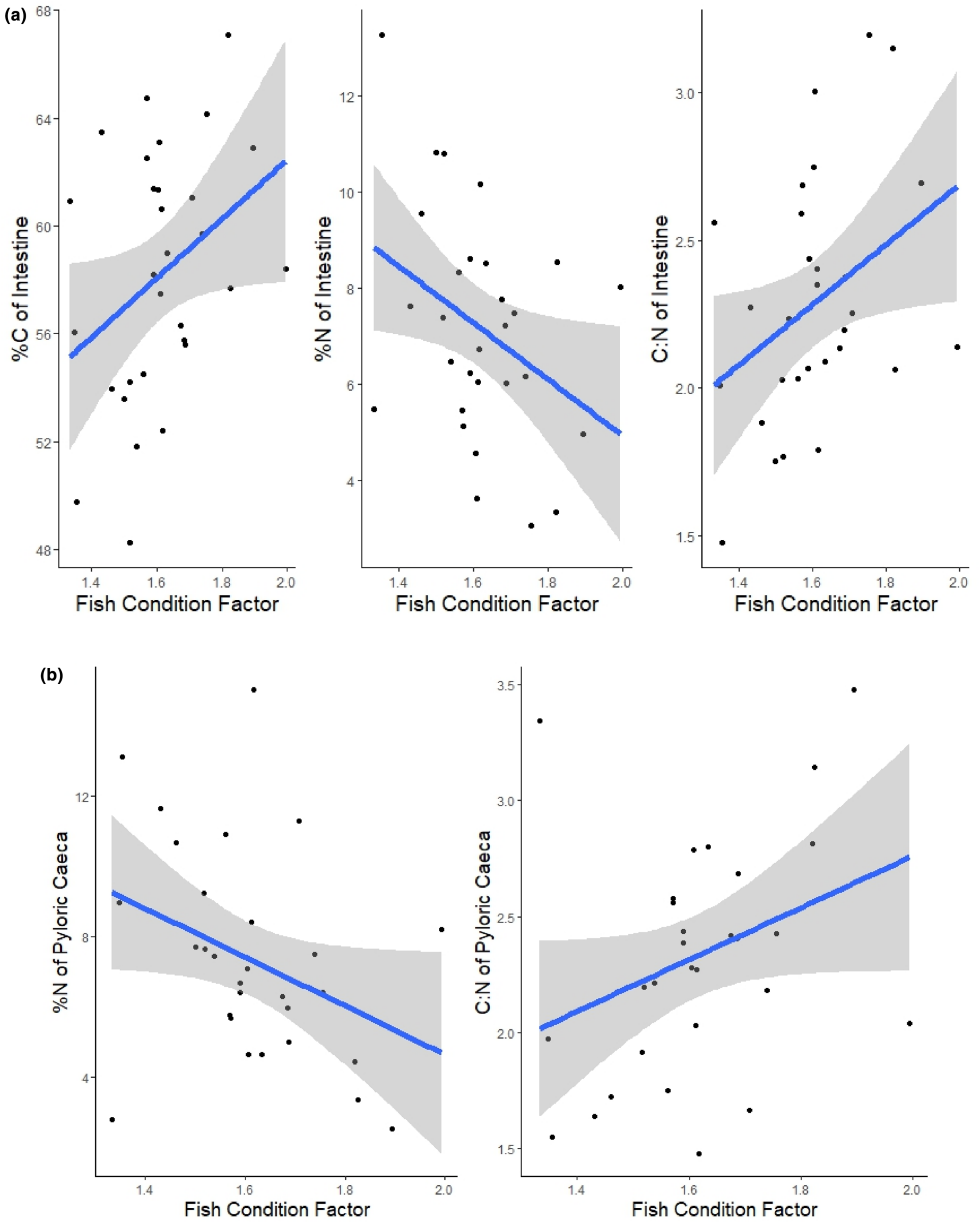
The relationship between condition factor and a. %C, %N and C:N ratio of the intestine, and b. %N and C:N of the pyloric caeca of 30 largemouth bass across collection sites from the Mississippi river. Condition factor was positively related to intestine %C, and C:N ratios and negatively related to intestine and pyloric caeca %N.

Across fish, condition factor was positively related to intestine %C, and C:N ratios and negatively related to intestine %N (%C *t*_28_ = 2.1, *p* = .05 *R* ^2^m = .13, *R* ^2^c = .13; %N *t*_28_ = −2.2, *p* = .04 *R* ^2^m = .14, *R* ^2^c = .14; C:N *t*_28_ = 2.2, *p* = .04 *R* ^2^m = .14, *R* ^2^c = .14, Figure 2, Table A2). Likewise, condition factor was marginally positively related to pyloric caeca C:N ratios and negatively related to pyloric caeca %N (%N *t*_29_ = −2.0, *p* = .05 *R* ^2^m = .14, *R* ^2^c = .17; C:N *t*_19.1_ = 1.7, *p* = .1 *R* ^2^m = .12, *R* ^2^c = .24, Figure 2, Table A2).

Six parasite genera from three phyla were found in more than 30% of the bass we dissected and were included in our statistical analyses. Three of these genera were found in the same three tissues (the stomach, pyloric caeca and intestines), but only two (*Leptorhynchoides* spp. and *Neoechinorhynchus* spp.) were found in high enough quantities for biomass analysis in multiple tissues. Additional details about all six parasites can be found in Table 1, and numbers of parasites found in each host are listed in Table A3.

**TABLE 1:** Parasites found in >30% of 30 largemouth bass taken from three collection sites within the Upper Mississippi River.

Genus	Phylum	Infection site in host	Functional feeding Group	% of bass infected
*Leptorhynchoides*	Acanthocephala	Stomach, Pyloric Caeca*, Intestines	Absorber	60%
*Neoechinorhynchus*	Acanthocephala	Stomach, Pyloric Caeca, Intestines*	Absorber	80%
*Contracaecum*	Nematoda	Visceral Mesentery	Encysted	80%
*Crepidostomum*	Platyhelminthes	Stomach, Pyloric Caeca*, Intestines*	Grazer	60%
*Posthodiplostomum*	Platyhelminthes	Liver	Encysted	95%

*Note*: Parasite genera, phylum, host organ, functional feeding group and percent of infected largemouth bass (in any tissue of the Bass) are shown. Absorbers are the same trophic level as their hosts, grazers are one level higher. The Asterix in host organ denotes the target organ for that parasite genera. Functional Feeding groups gathered from Chappell (1980).

**FIGURE 3:**
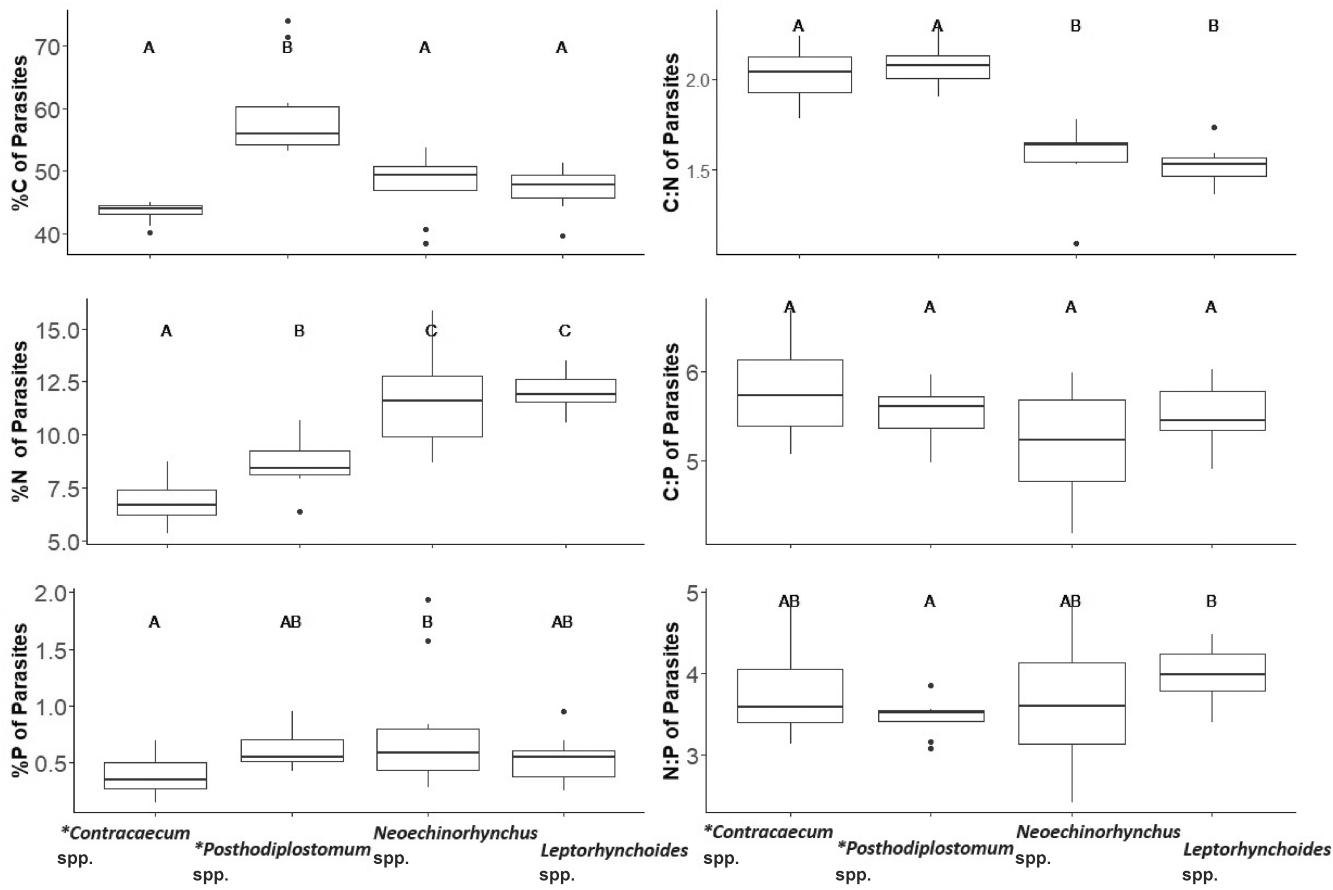
%C, %N, %P, C:N, C:P and N:P across parasite taxa collected from the digestive tracts of 30 largemouth bass. Boxes with the same letter associated with them are not significantly different according to Emmeans pairwise analysis with Tukey corrections. * indicates parasites found in their encysted life stage in the largemouth bass.

Parasite tissue %C, %N, %P, C:N and N:P differed among genera (%C *F*_(3,47)_ = 30.99, *p* < .001, *R* ^2^m = .65, *R* ^2^c = .65; %N *F*_(3,38.9)_ = 62.78, *p* < .001, *R* ^2^m = .78, *R* ^2^c = .82; %P *F*_(3,52.5)_ = 4.03, *p* = .01, *R* ^2^m = .13, *R* ^2^c = .40; C:N *F*_(3,47)_ = 49.7, *R* ^2^m = .75, *R* ^2^c = .75; *p* < .001, N:P *F*_(3,44.11)_ = 3.43, *p* = .02, *R* ^2^m = .13, *R* ^2^c = .47 Figure 3, Table A4). Specifically, we found that *Posthodiplostomum* spp., an encysted Platyhelminth in the liver, had a higher %C than other parasites and a higher %P than *Contracaecum* spp. (an encysted nematode in the visceral mesentery, Figure 3). However, both *Contracaecum* spp. *a*nd *Posthodiplostomum* spp. had lower %N (and higher C:N ratios) than *Leptorhynchoides* spp. and *Neoechinorhynchus* spp. (both absorbing acanthocephalans found in the stomach, pyloric caeca and intestines, Figure 2). parameters.

**FIGURE 4:**
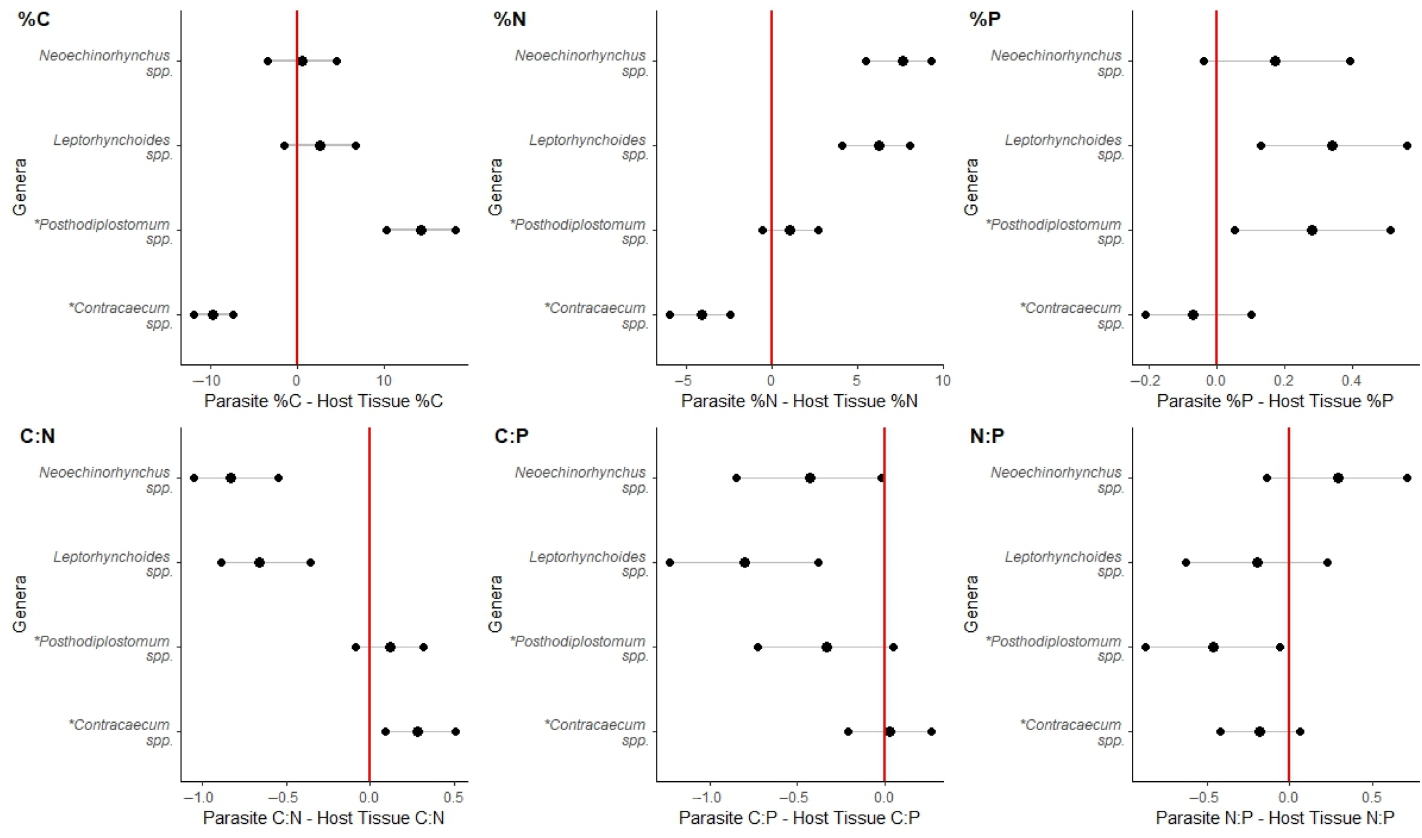
Confidence intervals of the mismatch of nutrient (%C, %N, %P, C:N, C:P and N:P) between parasite genera and the tissue they were found in from parasites collected from the digestive tracts of 25 largemouth bass collected from the Mississippi River. Centre points on bars are the means of the mismatch values of that parasite genera. * indicates that the parasite genera are found in its encysted life stage in the largemouth bass. Values that do not cross zero, were considered significantly different from their host tissue. Negative values indicate that the host tissue has a higher nutrient content or ratio, while positive numbers indicate that the parasite is a higher nutrient ratio or content.

Enough biomass was collected from four parasite genera to compare the mismatch between the stoichiometric parameters of the tissues of the parasites themselves to those of the tissues they were inhabiting. Parasites and host tissue were mismatched (%C *F*_3,47_ = 17.34, *p* ≤ .001, *R* ^2^m = .51, *R* ^2^c = .51; %N *F*_3,45.2_ = 29.52, *p* ≤ .001, *R* ^2^m = .63, *R* ^2^c = .73; %P *F*_3,44.17_ = 3.93 *p* = .01, *R* ^2^m = .15, *R* ^2^c = .31; C:N *F*_3,44.66_ = 25.04, *p* ≤ .001, *R* ^2^m = .61, *R* ^2^c = .70; C:P *F*_3,37.73_ = 5.0 *p* = .005, *R* ^2^m = .2, *R* ^2^c = .49; N:P *F*_3,41.22_ = 3.10, *p* = .04, *R* ^2^m = .16, *R* ^2^c = .30 Figure 4, Table A5). Positive confidence intervals that did not overlap with zero showed that *Posthodiplostomum* spp. had higher %C and %P than the host tissues it was found in. Negative confidence intervals show that *Posthodiplostomum* spp. had lower N:P than its host tissues. *Neoechinorhynchus* spp. and *Leptorhynchoides* spp. both had higher %N and lower C:N ratios than the host tissues they were found in. *Leptorhynchoides* spp. also had a higher %P and a lower C:P ratio than the host tissue it was found in. *Contracaecum* spp. had lower %C and %N but higher C:N ratio than its host tissues. All other stoichiometric comparisons of parasite to host tissue resulted in confidence intervals that overlapped with zero.

**FIGURE 5:**
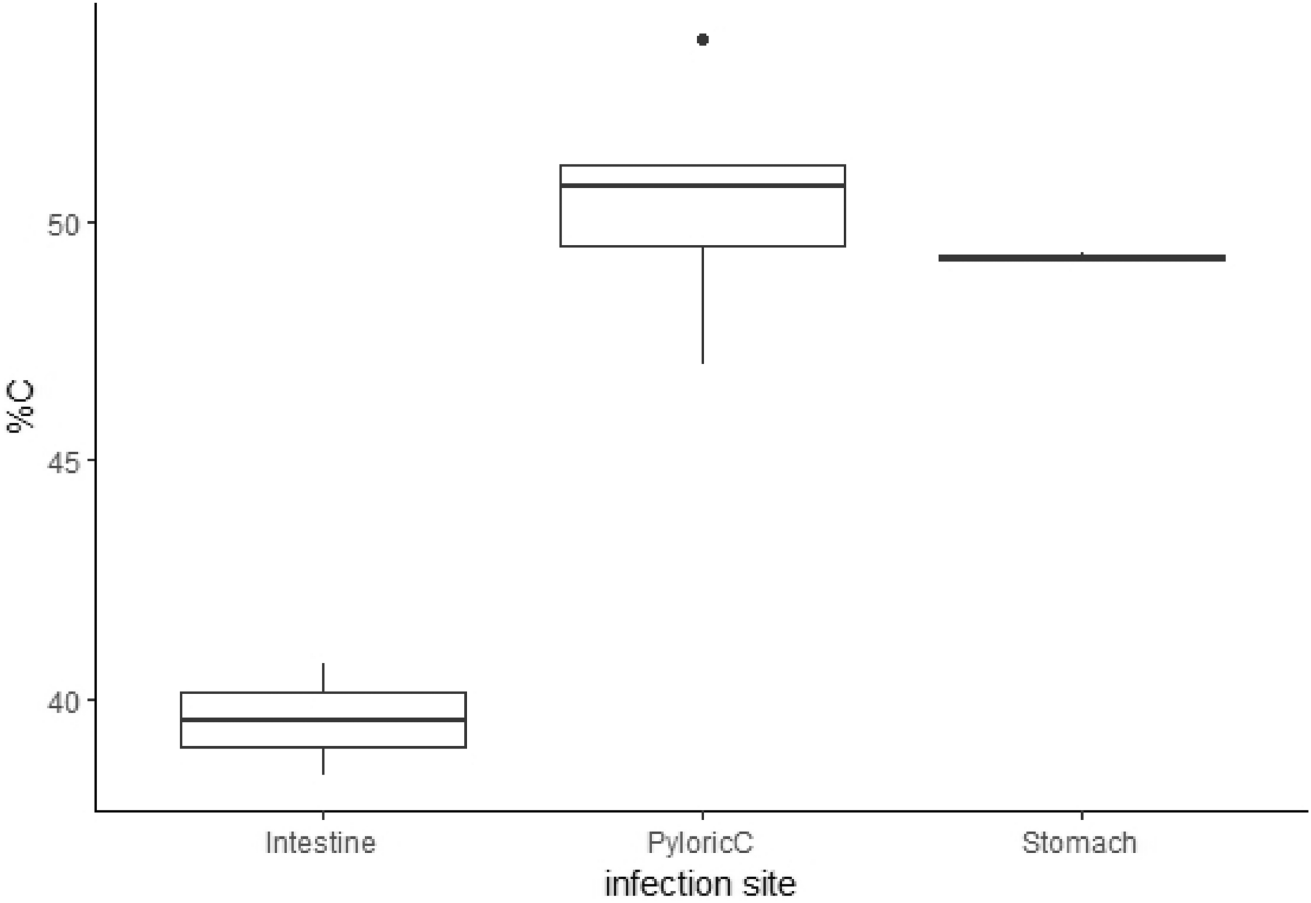
The %C in nine worms of *Leptorhynchoides* spp. across the organs that they were found in within the digestive tracts of seven Largemouth Bass collected from the Upper Mississippi River. These worms were found in three infection sites, the stomach, pyloric caeca and intestine, and had higher C content in the pyloric caeca than in the intestine.

We could only compare the stoichiometric parameters of the acanthocephalan absorbers (*Leptorhynchoides* spp. and *Neoechinorhynchus* spp.) across the stoichiometric parameters of different infection sites because they were the only parasites that occurred in high enough prevalence and biomass for nutrient analysis across multiple infection sites. *Leptorhynchoides* spp. worms from different infection sites varied in their %C, with worms in the intestines having significantly less C than worms found in the pyloric caeca or stomach (*F*_2,2.9_ = 24.30, *p* = .02, *R* ^2^m = .83, *R* ^2^c = .86, Figure 5, Table A6). No other significant relationships between the stoichiometric parameters of a parasite and host organs were observed.

### 3.2 Parasite load

**FIGURE 6:**
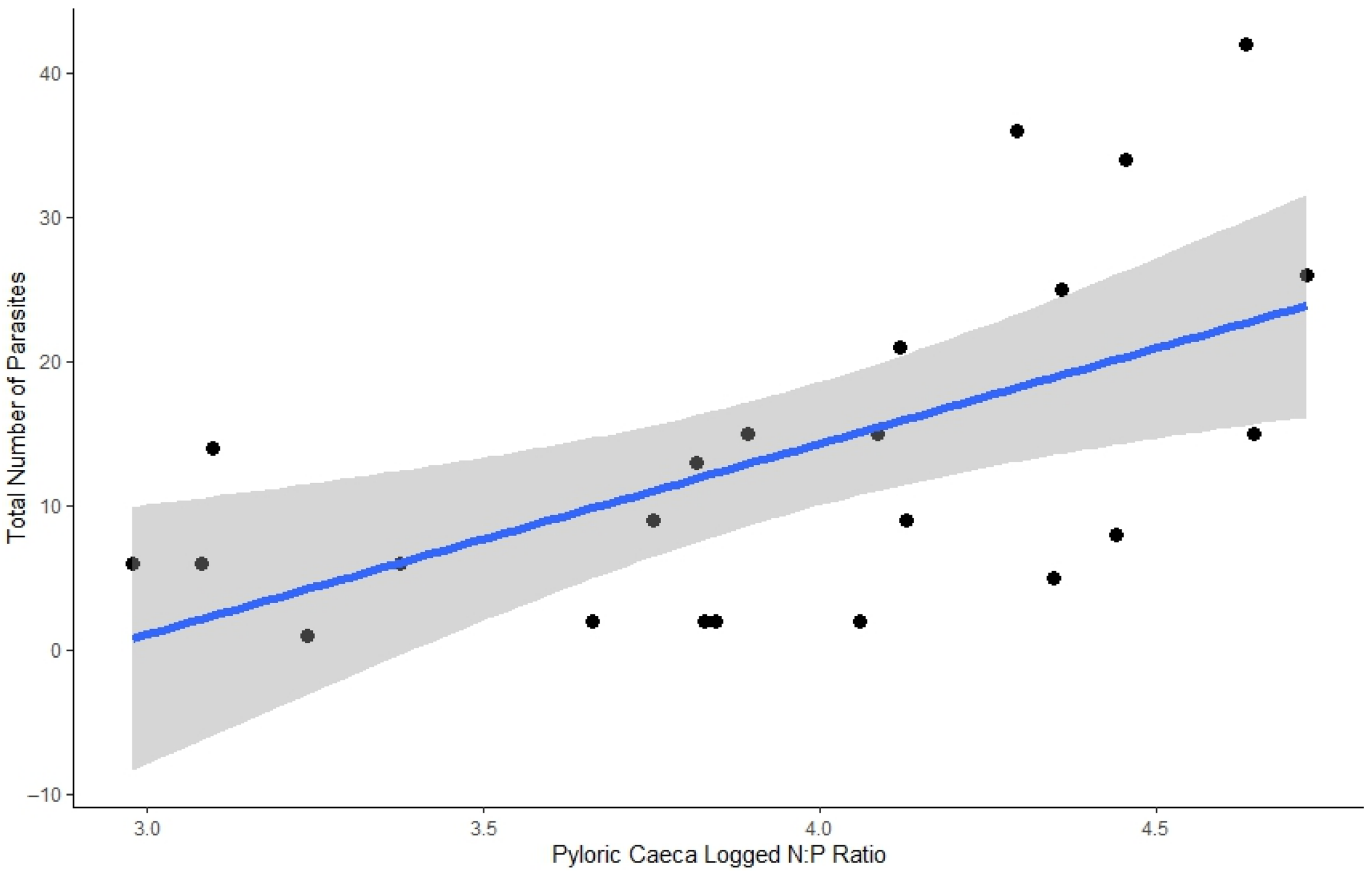
The total number of parasitic worms found within the pyloric caeca of 23 largemouth bass collected from the Upper Mississippi River increased as a function of the N:P of the pyloric caeca.

The abundance of each parasite genera within an organ was best explained by the null model when compared with models including organ stoichiometry, the mismatch between parasite and organ stoichiometry and condition factor (Tables A6,A7, and A8). However, when all parasites within an organ were combined (when we examined the size of the entire infracommunity within each organ), model selection indicated that the model including the N:P of the pyloric caeca was the best model for this response. Within this model, the N:P ratio was positively correlated with infracommunity size (Estimate = 0.79, SE = 0.32, *p* value = .02, *R* ^2^m = .21, *R* ^2^c = .33 Figure 6, Table A6).

## 4 DISCUSSION

Ecosystem nutrient availability can affect the prevalence and severity of parasitism through a variety of mechanisms (Aalto et al., 2015). The effect of host diet nutrient content on parasitism has been examined at scales from the individual (Frost et al., 2008; Narr & Frost, 2016) to the ecosystem level (Johnson et al., 2007; Mischler et al., 2016; Narr et al., 2019), but our study suggests that an even finer level of resolution can help illuminate the mechanisms responsible for this pathway. We show that host stoichiometry varies within hosts (i.e. among tissues and organs, Figure 1) and across hosts (as a function of host condition), and this variation is associated with variation in parasite abundance. The C:N ratios of fish intestines and pyloric caeca increased with fish condition, but actively feeding parasites had consistently lower C:N ratios than their hosts (Figures 2 and 3), suggesting the potential for N limitation of actively feeding parasites in hosts in good condition. Consistent with the potential for N limitation of these parasites, we observed a positive relationship between the N:P ratio of the pyloric caeca and the abundance of parasites in this tissue. These results suggest that shifts in nutrient availability within a host's environment exert the strongest effects on parasites of tissues involved in nutrient absorption. Below, we explore the potential causes and consequences of the variation in host and parasite tissue stoichiometry and the relationship between pyloric caeca N:P and infracommunity that we observed.

### 4.1 Organ and parasite stoichiometry

While the role of an infection site in shaping the nutritional environment of a parasite is widely and intuitively acknowledged in the field of parasitology, stoichiometric variation within a host is routinely ignored in the application of ecological stoichiometry to host–parasite interactions. In many host–parasites systems, this variation may have been overlooked because the host is too small for traditional nutrient analysis on individual infection sites (e.g. *Daphnia*‐parasite, or phytoplankton systems), and it is possible that the assumption of a relatively homogenous distribution of nutrients within hosts is justifiable in, for example, single‐celled hosts. However, our data from a relatively large vertebrate host clearly show significant stoichiometric variation among infection sites that is consistent across hosts (Figure 1). The nutrient content of individual fish organs has, to our knowledge, not been previously compared, but the differences that we observed are consistent with the roles of these organs in digestion. We suspect that the relatively low C:N (and high %N) of the stomach and liver may be due to their respective production of pepsin and urea. Pepsin and urea are both relatively high in nitrogen and not found elsewhere in the digestive tract (Kong et al., 1998; Sarbahi, 1951). If bass parasites can capitalize on N‐rich compounds like pepsin, competition for these resources from parasites may help explain patterns in parasite virulence, and this stoichiometric variation within hosts may have played a role in the evolution of site‐specific parasites.

One of the goals of applying ecological stoichiometry to host–parasite systems is to connect nutrients in a host's environment to the nutrients contained within a host and by extension nutrients in a parasite's environment. Because our study focuses on mobile hosts that can encounter variation in resource availability, this should result in variability in resources available to individual parasites infecting different hosts. In largemouth bass, we expected that natural variation in their diets would influence host condition and cause the stoichiometry of the primary site of nutrient absorption, the pyloric caeca (Buddington & Diamond, 1986), to vary across hosts more than other organs. Consistent with this prediction, the stoichiometry of both fish intestines and pyloric caeca were related to host condition while the stoichiometry of other tissues was not (Figure 2). The consequences of this variation for the success of parasites living in this organ depend, in large part, on the nutritional needs of the parasite.

Like any other animal, the nutritional needs of a parasite can be estimated based on the stoichiometry of their tissues (Reiners, 1986). Our study contributes to a small but growing understanding of these needs by quantifying differences in the stoichiometry of parasite genera. Just as the stoichiometry of infection sites differed primarily in terms of C, N and C:N ratios, we found that parasite genera exhibited more differences in the stoichiometric parameters involving C and N than P (Figure 3). This result suggests that the nutritional needs of the four parasites whose stoichiometry we measured did not consistently differ in their P requirements. The resource ratio hypothesis suggests that, when organisms compete for resources, the organism capable of surviving on the lowest level of the most limiting resource will win (Tilman, 1982). Given the limited variability in the P content of these parasites, we suggest that C or N availability may be more likely to dictate the outcome of competition between these parasites within a host. Of the parasites whose stoichiometry we measured, we anticipate that competition would be most intense between the two actively feeding parasites, especially when they inhabit the same tissue. Consistent with previous work (Mischler et al., 2016; Paseka & Grunberg, 2019), we found that parasites at non‐reproductive stages (i.e. encysted *Contracaecum* spp. and *Posthodiplostomum* spp.) had higher C:N ratios than those in actively feeding life stages. We suspect these differences are a result of the structural requirements of encysted parasites. *Posthodiplostomum* spp., has a nitrogen‐rich tegument, but this is thinner during the encysted metacercaria stage possibly because some layers of the tegument are sloughed off to form the parasite layer of the cyst (Hunter & Hunter, 1940). Our analysis did not include the tissue forming the outer layers of the cyst, so the reduced tegument could result in a higher C:N ratio (Fried & Graczyk, 1997). Encysted parasites may also have lower N content than actively feeding stages because of the protection their cysts and locations provide. When present in the stomach, pyloric caeca, or intestines, actively feeding parasites must counteract powerful proteinases, secreted by the host for the purposes of digestion and immune response, through the production of proteinases inhibitors (Knox, 2007). These proteinase inhibitors are themselves protein rich (Turk et al., 2008), resulting in the need for a higher %N.

In addition to stoichiometric differences across genera, our study provides additional insight into the stoichiometric flexibility of parasites. We achieved this by quantifying stoichiometric differences within genera, across infection sites, or across organs of varying nutrient content. We found that *Leptorhyncoides* spp. worms in the intestine had lower C than those occupying the stomach or the pyloric caeca (Figure 5). We suspect that this difference is related to the energetic needs of the worm as it travels through its host. The target infection site for *Leptorhyncoides* spp. in largemouth bass is the pyloric caeca, but the worms can establish almost anywhere in the digestive system and move to the pyloric caeca over the course of several weeks (Leadabrand & Nickol, 1993). The lower C of *Leptorhyncoides* spp. in the intestine may be a sign that this worm relies on energy stores while they make their way to the caeca. Alternatively, it is possible that being in a suboptimal portion of the digestive tract (the intestines) may exert an energetic cost which is reflected in the lower C of worms.

### 4.2 Parasite load

**FIGURE A1:**
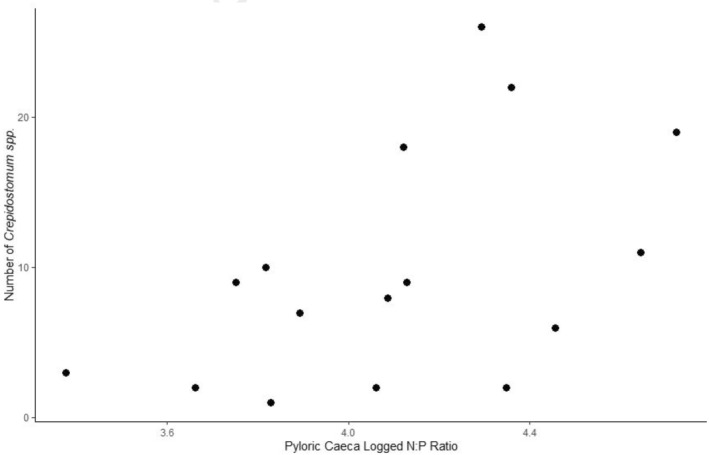
The *Crepidostomum* spp. worms found within the pyloric caeca of 23 largemouth bass collected from the Upper Mississippi River increased as a function of the N:P of the pyloric caeca.

We demonstrated substantial stoichiometric variation across infection sites and parasitic genera (both across and within genera), but what are the consequences of this variation on parasite load? We found the N:P ratio of the pyloric caeca was positively correlated with the abundance of parasites in that organ. There are a few non‐mutually exclusive hypotheses that may explain why the size of the parasite infracommunity of the pyloric caeca increased with organ N:P ratios. This infracommunity was primarily (on average ~74%) composed of the trematode *Crepidostomum* spp. As a result, while the null model was better than pyloric caeca N:P ratios at describing variation *Crepidistomum* load, the relationship between the load of *Crepidistomum* spp. and pyloric caeca N:P ratios (estimate = 1.08) was qualitatively similar to that describing total parasite load and pyloric caeca N:P (estimate = 0.79, Figure A1). While we were unable to obtain nutrient contents for *Crepidostomum* spp., other studies, like ours, have found higher N in active feeding parasites relative to encysted ones (Paseka & Grunberg, 2019), and our data suggest that actively feeding parasites have lower %C and C:N ratios and higher %N than the tissues they occupy (Figure 6). These trends suggest that N could limit actively feeding parasitic worms, and higher parasitic loads in the pyloric caeca of largemouth bass could result from increases in N availability in the host environment. However, the role of P in this relationship remains unclear. It is possible that reducing the availability of P relative to N enabled *Crepidostomum* spp. to outcompete parasites like *Leptorhynchoides* spp. who may have higher P requirements, but we are unable to test this hypothesis with our data. We suspect that additional processes (outlined below) may be responsible for the strength of this relationship between infracommunity load and pyloric caeca N:P.

The positive relationship between infracommunity load and pyloric caeca N:P ratios may be an effect of the parasite, rather than the host. At high biomasses, trematodes in snail hosts can enhance ecosystem availability of N due to changes in host assimilation and excretion rates (Mischler et al., 2016). Given that a trematode comprised most of the infracommunity in the pyloric caeca, it is possible that similar shifts could simultaneously alter the nutrients available to hosts while increasing parasite loads. Likewise, a positive relationship between the N:P of rock pools and the prevalence of a parasite in *Daphnia* was consistent with parasite‐induced shifts in host nutrient excretion (Narr et al., 2019). More direct effects of parasites could also be responsible for the nutrient ratios in the pyloric caeca. The change in N:P may indicate an immune response of the tissue to high parasitic load. In other studies, Acanthocephalans, specifically *Leptorhynchoides thecatus*, caused pyloric caeca muscle thickening and infiltration of the tissue by leucocytes and neutrophils (de Buron & Nickol, 1994; Venard & Warfel, 1953). In other fish, muscle tissue is relatively high in %N compared to the entire fish (Vrede et al., 2011). If this effect is indeed caused by the infracommunity, changes in the N:P of the pyloric caeca may be a useful indicator of immune response in fish.

The mismatch between the elemental composition of parasite and host is also expected to have a strong influence on the success of a parasite (Aalto et al., 2015). Parasites with stoichiometries that are like those of their host are generally expected to have higher fecundity and infection rates than parasites that differ from their host. Despite the intuitive logic of this comparison, very few studies have explicitly measured this mismatch (but see, Narr & Krist, 2015; Frenken et al., 2021), so we have little data to determine how useful this mismatch is in predicting outcomes for the parasite. We examined this possibility by comparing stoichiometric mismatches to the parasite load in each tissue, and we were unable to detect an effect of this mismatch on the abundances of the parasites we examined. It is possible that we did not observe this relationship because the parasites whose stoichiometry we were able to measure likely do not feed directly on the tissues themselves. Consistent with this possibility, the strongest relationship we observed between the stoichiometry of host tissues and parasite abundance in our system was in an infracommunity comprised primarily of a grazing parasite. However, it is also possible that more statistical power is needed to detect these trends.

### 4.3 Conclusions

Our results add to a growing body of research showing important relationships between host nutrient content and parasites. Theory predicts that stoichiometric variation within and across species drives the outcome of species interactions (Sterner & Elser, 2002). Understanding the mechanisms that drive this variation within and among parasite genera and the tissues they infect will help us predict their effect on the outcomes of the parasite–host interaction. We showed that the condition of a host is related to variation in the nutrient content of some (but not all) of its infection sites, and that related stoichiometric variation is relevant to the size of the parasite infracommunties occupying that infection site. We also confirmed that different parasite genera had different stoichiometric traits. These observations provide a step forward in our ability to predict the effects of nutrient additions on parasitism, especially using the framework of ecological stoichiometry.

## AUTHOR CONTRIBUTIONS

**Adrienne Stanley:** Conceptualization (equal); data curation (lead); formal analysis (equal); investigation (equal); methodology (equal); visualization (equal); writing – original draft (lead); writing – review and editing (supporting). **Shaley Valentine:** Data curation (supporting); resources (equal); writing – review and editing (equal). **Charlotte F. Narr:** Conceptualization (equal); formal analysis (equal); investigation (equal); methodology (equal); supervision (lead); writing – original draft (supporting); writing – review and editing (lead).

## FUNDING INFORMATION

There are no funders to report for this submission.

## CONFLICT OF INTEREST STATEMENT

The authors have no conflict of interest to declare.

## Data Availability

The data that support the findings of this study are openly available in figshare at https://figshare.com/s/44273215043862cc1c80, reference number 10.6084/m9.figshare.23532903.

## APPENDIX A

**TABLE A1:** Results of Tukey post hoc tests comparing the %C, %N, %P, C:N, C:P, and N:P of organs from 30 largemouth bass collected at three sites in the Upper Mississippi.

Organs Compared	%C	%N	%P	C:N	C:P	N:P
Intestine × Liver	**Diff = 3.64**	**Diff = −3.15**	Diff = −0.01	**Diff = 4.52**	Diff = 114	Diff = −10.2
	***p* = .003**	***p* = <.001**	*p* = .99	***p* = .0001**	*p* = .16	*p* = .24
Intestine × Pyloric C.	Diff = −1.40	Diff = −0.09	Diff = 0.09	Diff = −0.84	Diff = −101	Diff = −7.13
	*p* = .56	*p* = .99	*p* = .12	*p* = .88	*p* = .31	*p* = .54
Intestine × Stomach	**Diff = 9.8**	**Diff = −5.30**	Diff = −0.09	**Diff = 6.05**	**Diff = 209**	Diff = −7.41
	***p* = <.001**	***p* = <.001**	*p* = .12	***p* = .0001**	***p* = .0003**	*p* = .51
Intestine × Viscera	**Diff = 4.47**	**Diff = −2.54**	Diff = 0.01	Diff = 4.13	Diff = 118	Diff = −5.59
	***p*= .0001**	***p*= <.0001**	*p*= .99	*p*= .0001	*p*= .11	*p*= .76
Liver × Pyloric C.	**Diff = −5.05**	**Diff = 3.06**	Diff = 0.11	**Diff = −5.36**	**Diff = −214**	Diff = 3.08
	***p* = <.001**	***p* = <.001**	*p* = .08	***p* = <.001**	***p* = .0003**	*p* = .97
Liver × Stomach	**Diff = 6.2**	**Diff = −2.15**	Diff = −0.08	Diff = 1.53	Diff = 95.5	Diff = 2.80
	***p* = <.001**	***p* = .0001**	*p* = .29	*p* = .50	*p* = .33	*p* = .94
Liver × Viscera	Diff = 0.83	Diff = 0.61	Diff = 0.03	Diff = −0.39	Diff = 4.28	Diff = 4.61
	*p* = .92	*p* = .68	*p* = .97	*p* = .99	*p* = 1.00	*p* = .88
Pyloric C. × Stomach	**Diff = 11.2**	**Diff = −5.21**	**Diff = −0.19**	**Diff = 6.89**	**Diff = 310**	Diff = −0.28
	***p* = <.001**	***p* = <.001**	***p* = <.001**	***p* = <.001**	***p* = <.001**	*p* = 1.00
Pyloric C. × Viscera	**Diff = 5.88**	**Diff = −2.45**	Diff = −0.08	**Diff = 4.97**	**Diff = 219**	Diff = 1.53
	***p* = <.001**	***p* = <.001**	*p* = .26	***p* = <.001**	***p* = .0001**	*p* = .99
Stomach × Viscera	**Diff = −5.36**	**Diff = 2.76**	Diff = 0.11*	Diff = −1.92	Diff = −91.2	Diff = 1.81
	***p* = <.001**	***p* = <.001**	*p* = .06*	*p* = .23	*p* = .33	*p* = .99

*Note*: Diff is the estimated difference of the mean given between the organs, and *p* is the significance of the difference. Values shown in bold indicate significant (*p*<.05) differences. Starred values indicate marginally significant differences (.05< *p* <.10).

**TABLE A2:** Results of linear mixed models comparing the nutrient content of specific organs to the condition factors of the fish.

Organ	%C	%N	%P	C:N	C:P	N:P
Liver	Estimate: 0.58	Estimate: −0.04	Estimate: −0.12	Estimate: 0.004	Estimate: 0.43	Estimate: 0.43
	*t*_19.4_ = 0.26	*t*_23_ = −0.03	*t*_23_ = −0.52	*t*_23_ = 0.03	*t*_23_ = 0.71	*t*_23_ = 0.71
	*p* = .80	*p* = .97	*p* = .61	*p* = .98	*p* = .48	*p* = .49
Stomach	Estimate: −5.6	Estimate: −0.20	Estimate:0.12	Estimate: −0.1	Estimate: −0.44	Estimate: −0.34
	*t*_28_ = −1.1	*t*_28_ = −0.134	*t*_28_ = 0.5	*t*_28_ = −0.47	*t*_28_ = −0.85	*t*_28_ = −0.78
	*p* = .30	*p* = .89	*p* = .62	*p* = .64	*p* = .40	*p* = .44
Pyloric Caeca	Estimate: 9.28	**Estimate: −6.91**	Estimate: 0.18	Estimate: 1.0	Estimate: −0.19	Estimate: −1.2
	*t*_29_ = 1.5	***t***_**29**_ **= −2.0**	*t*_29_ = 0.68	*t*_19.1_ = 1.7	*t*_29_ = 1.7	*t*_18.8_ = 1.6
	*p* = .15	***p* = .05**	*p* = .50	*p* = .1*	*p* = .8	*p* = .13
Intestine	**Estimate: 11**	**Estimate: −5.9**	Estimate: 0.02	**Estimate: 1.0**	Estimate: 0.11	Estimate: −0.58
	***t***_**28**_ **= 2.06**	***t***_**28**_ **= −2.2**	*t*_28_ = 0.07	***t***_**28**_ **= 2.2**	*t*_28_ = 0.14	*t*_23.2_ = −0.65
	***p* = .05**	***p* = .03**	*p* = .94	***p* = .034**	*p* = .89	*p* = .52

*Note*: Bold font denotes significant results, asterisk denotes marginal significance.

**TABLE A3:** A list of parasites found in the digestive tracts of 30 Largemouth Bass collected from the Mississippi River.

Fish Id	Parasite	Infection Site	Number of Worms
354	*Contracaecum* spp.	Viscera	116
	*Posthodiplostomum* spp.	Liver	429
	*Leptorhynchoides* spp.	Stomach	0
		Pyloric Caeca	0
		Intestine	0
	*Neoechinorhynchus* spp.	Stomach	0
		Pyloric Caeca	0
		Intestine	0
	*Crepidostomum* spp.	Stomach	0
		Pyloric Caeca	3
		Intestine	0
	*Spinitectus* spp.	Stomach	6
		Pyloric Caeca	0
		Intestine	3
401	*Contracaecum* spp.	Viscera	0
	*Posthodiplostomum* spp.	Liver	0
	*Leptorhynchoides* spp.	Stomach	0
		Pyloric Caeca	0
		Intestine	0
	*Neoechinorhynchus* spp.	Stomach	2
		Pyloric Caeca	0
		Intestine	5
	*Crepidostomum* spp.	Stomach	0
		Pyloric Caeca	0
		Intestine	0
	*Spinitectus* spp.	Stomach	0
		Pyloric Caeca	0
		Stomach	0
417	*Contracaecum* spp.	Viscera	35
	*Posthodiplostomum spp*.	Liver	190
	*Leptorhynchoides* spp.	Stomach	0
		Pyloric Caeca	0
		Intestine	0
	*Neoechinorhynchus* spp.	Stomach	0
		Pyloric Caeca	0
		Intestine	3
	*Crepidostomum* spp.	Stomach	16
		Pyloric Caeca	9
		Intestine	8
	*Spinitectus* spp.	Stomach	0
		Pyloric Caeca	0
		Stomach	0
359	*Contracaecum* spp.	Viscera	10
	*Posthodiplostomum* spp.	Liver	256
	*Leptorhynchoides* spp.	Stomach	0
		Pyloric Caeca	6
		Intestine	0
	*Neoechinorhynchus* spp.	Stomach	1
		Pyloric Caeca	0
		Intestine	7
	*Crepidostomum* spp.	Stomach	0
		Pyloric Caeca	0
		Intestine	0
	*Spinitectus* spp.	Stomach	0
		Pyloric Caeca	0
		Stomach	0
356	*Contracaecum* spp.	Viscera	71
	*Posthodiplostomum* spp.	Liver	608
	*Leptorhynchoides* spp.	Stomach	3
		Pyloric Caeca	0
		Intestine	5
	*Neoechinorhynchus* spp.	Stomach	0
		Pyloric Caeca	0
		Intestine	0
	*Crepidostomum* spp.	Stomach	0
		Pyloric Caeca	0
		Intestine	0
	*Spinitectus* spp.	Stomach	0
		Pyloric Caeca	0
		Intestine	0
171	*Contracaecum* spp.	Viscera	18
	*Posthodiplostomum* spp.	Liver	577
	*Leptorhynchoides* spp.	Stomach	0
		Pyloric Caeca	8
		Intestine	2
	*Neoechinorhynchus* spp.	Stomach	13
		Pyloric Caeca	0
		Intestine	5
	*Crepidostomum* spp.	Stomach	0
		Pyloric Caeca	0
		Intestine	0
	*Spinitectus* spp.	Stomach	0
		Pyloric Caeca	0
		Intestine	0
162	*Contracaecum* spp.	Viscera	55
	*Posthodiplostomum* spp.	Liver	68
	*Leptorhynchoides* spp.	Stomach	0
		Pyloric Caeca	0
		Intestine	0
	*Neoechinorhynchus* spp.	Stomach	0
		Pyloric Caeca	0
		Intestine	5
	*Crepidostomum* spp.	Stomach	0
		Pyloric Caeca	2
		Intestine	0
	*Spinitectus* spp.	Stomach	0
		Pyloric Caeca	0
		Intestine	0
352	*Contracaecum* spp.	Viscera	0
	*Posthodiplostomum* spp.	Liver	258
	*Leptorhynchoides* spp.	Stomach	0
		Pyloric Caeca	0
		Intestine	6
	*Neoechinorhynchus* spp.	Stomach	1
		Pyloric Caeca	0
		Intestine	5
	*Crepidostomum* spp.	Stomach	0
		Pyloric Caeca	0
		Intestine	0
	*Spinitectus* spp.	Stomach	0
		Pyloric Caeca	0
		Intestine	0
357	*Contracaecum* spp.	Viscera	0
	*Posthodiplostomum* spp.	Liver	354
	*Leptorhynchoides* spp.	Stomach	0
		Pyloric Caeca	0
		Intestine	0
	*Neoechinorhynchus* spp.	Stomach	0
		Pyloric Caeca	0
		Intestine	0
	*Crepidostomum* spp.	Stomach	0
		Pyloric Caeca	0
		Intestine	0
	*Spinitectus* spp.	Stomach	0
		Pyloric Caeca	0
		Intestine	0
163	*Contracaecum* spp.	Viscera	60
	*Posthodiplostomum* spp.	Liver	572
	*Leptorhynchoides* spp.	Stomach	0
		Pyloric Caeca	0
		Intestine	0
	*Neoechinorhynchus* spp.	Stomach	0
		Pyloric Caeca	8
		Intestine	1
	*Crepidostomum* spp.	Stomach	0
		Pyloric Caeca	7
		Intestine	7
	*Spinitectus* spp.	Stomach	0
		Pyloric Caeca	0
		Intestine	0
166	*Contracaecum* spp.	Viscera	70
	*Posthodiplostomum* spp.	Liver	349
	*Leptorhynchoides* spp.	Stomach	0
		Pyloric Caeca	3
		Intestine	0
	*Neoechinorhynchus* spp.	Stomach	0
		Pyloric Caeca	0
		Intestine	3
	*Crepidostomum* spp.	Stomach	0
		Pyloric Caeca	2
		Intestine	0
	*Spinitectus* spp.	Stomach	0
		Pyloric Caeca	0
		Intestine	0
157	*Contracaecum* spp.	Viscera	50
	*Posthodiplostomum* spp.	Liver	1679
	*Leptorhynchoides* spp.	Stomach	0
		Pyloric Caeca	0
		Intestine	0
	*Neoechinorhynchus* spp.	Stomach	0
		Pyloric Caeca	3
		Intestine	25
	*Crepidostomum* spp.	Stomach	9
		Pyloric Caeca	18
		Intestine	6
	*Spinitectus* spp.	Stomach	0
		Pyloric Caeca	0
		Intestine	0
172	*Contracaecum* spp.	Viscera	129
	*Posthodiplostomum* spp.	Liver	165
	*Leptorhynchoides* spp.	Stomach	1
		Pyloric Caeca	28
		Intestine	52
	*Neoechinorhynchus* spp.	Stomach	5
		Pyloric Caeca	0
		Intestine	0
	*Crepidostomum* spp.	Stomach	0
		Pyloric Caeca	6
		Intestine	0
	*Spinitectus* spp.	Stomach	0
		Pyloric Caeca	0
		Intestine	0
405	*Contracaecum* spp.	Viscera	6
	*Posthodiplostomum* spp.	Liver	109
	*Leptorhynchoides* spp.	Stomach	0
		Pyloric Caeca	0
		Intestine	0
	*Neoechinorhynchus* spp.	Stomach	2
		Pyloric Caeca	7
		Intestine	28
	*Crepidostomum* spp.	Stomach	0
		Pyloric Caeca	8
		Intestine	0
	*Spinitectus* spp.	Stomach	0
		Pyloric Caeca	0
		Intestine	0
156	*Contracaecum* spp.	Viscera	14
	*Posthodiplostomum* spp.	Liver	574
	*Leptorhynchoides* spp.	Stomach	0
		Pyloric Caeca	0
		Intestine	0
	*Neoechinorhynchus* spp.	Stomach	0
		Pyloric Caeca	1
		Intestine	14
	*Crepidostomum* spp.	Stomach	0
		Pyloric Caeca	1
		Intestine	0
	*Spinitectus* spp.	Stomach	0
		Pyloric Caeca	0
		Intestine	0
210	*Contracaecum* spp.	Viscera	67
	*Posthodiplostomum* spp.	Liver	1029
	*Leptorhynchoides* spp.	Stomach	3
		Pyloric Caeca	34
		Intestine	20
	*Neoechinorhynchus* spp.	Stomach	0
		Pyloric Caeca	0
		Intestine	5
	*Crepidostomum* spp.	Stomach	2
		Pyloric Caeca	91
		Intestine	0
	*Spinitectus* spp.	Stomach	0
		Pyloric Caeca	0
		Intestine	0
150	*Contracaecum* spp.	Viscera	1
	*Posthodiplostomum* spp.	Liver	0
	*Leptorhynchoides* spp.	Stomach	0
		Pyloric Caeca	1
		Intestine	0
	*Neoechinorhynchus* spp.	Stomach	0
		Pyloric Caeca	1
		Intestine	2
	*Crepidostomum* spp.	Stomach	0
		Pyloric Caeca	0
		Intestine	0
	*Spinitectus* spp.	Stomach	0
		Pyloric Caeca	0
		Intestine	0
212	*Contracaecum* spp.	Viscera	5
	*Posthodiplostomum* spp.	Liver	0
	*Leptorhynchoides* spp.	Stomach	6
		Pyloric Caeca	14
		Intestine	3
	*Neoechinorhynchus* spp.	Stomach	0
		Pyloric Caeca	0
		Intestine	0
	*Crepidostomum* spp.	Stomach	0
		Pyloric Caeca	0
		Intestine	0
	*Spinitectus* spp.	Stomach	0
		Pyloric Caeca	0
		Intestine	0
511	*Contracaecum* spp.	Viscera	0
	*Posthodiplostomum* spp.	Liver	76
	*Leptorhynchoides* spp.	Stomach	0
		Pyloric Caeca	0
		Intestine	5
	*Neoechinorhynchus* spp.	Stomach	0
		Pyloric Caeca	7
		Intestine	1
	*Crepidostomum* spp.	Stomach	0
		Pyloric Caeca	19
		Intestine	0
	*Spinitectus* spp.	Stomach	0
		Pyloric Caeca	0
		Intestine	0
91	*Contracaecum* spp.	Viscera	0
	*Posthodiplostomum* spp.	Liver	0
	*Leptorhynchoides* spp.	Stomach	0
		Pyloric Caeca	0
		Intestine	1
	*Neoechinorhynchus* spp.	Stomach	1
		Pyloric Caeca	0
		Intestine	11
	*Crepidostomum* spp.	Stomach	2
		Pyloric Caeca	42
		Intestine	1
	*Spinitectus* spp.	Stomach	0
		Pyloric Caeca	0
		Intestine	0
83	*Contracaecum* spp.	Viscera	0
	*Posthodiplostomum* spp.	Liver	0
	*Leptorhynchoides* spp.	Stomach	0
		Pyloric Caeca	2
		Intestine	0
	*Neoechinorhynchus* spp.	Stomach	0
		Pyloric Caeca	0
		Intestine	6
	*Crepidostomum* spp.	Stomach	0
		Pyloric Caeca	0
		Intestine	0
	*Spinitectus* spp.	Stomach	0
		Pyloric Caeca	0
		Intestine	0
149	*Contracaecum* spp.	Viscera	6
	*Posthodiplostomum* spp.	Liver	153
	*Leptorhynchoides* spp.	Stomach	0
		Pyloric Caeca	0
		Intestine	4
	*Neoechinorhynchus* spp.	Stomach	0
		Pyloric Caeca	3
		Intestine	4
	*Crepidostomum* spp.	Stomach	0
		Pyloric Caeca	10
		Intestine	1
	*Spinitectus* spp.	Stomach	0
		Pyloric Caeca	0
		Intestine	0
146	*Contracaecum* spp.	Viscera	9
	*Posthodiplostomum* spp.	Liver	149
	*Leptorhynchoides* spp.	Stomach	4
		Pyloric Caeca	2
		Intestine	5
	*Neoechinorhynchus* spp.	Stomach	0
		Pyloric Caeca	4
		Intestine	0
	*Crepidostomum* spp.	Stomach	0
		Pyloric Caeca	0
		Intestine	0
	*Spinitectus* spp.	Stomach	0
		Pyloric Caeca	0
		Intestine	0
223	*Contracaecum* spp.	Viscera	0
	*Posthodiplostomum* spp.	Liver	0
	*Leptorhynchoides* spp.	Stomach	0
		Pyloric Caeca	0
		Intestine	0
	*Neoechinorhynchus* spp.	Stomach	0
		Pyloric Caeca	0
		Intestine	4
	*Crepidostomum* spp.	Stomach	2
		Pyloric Caeca	0
		Intestine	0
	*Spinitectus* spp.	Stomach	3
		Pyloric Caeca	0
		Intestine	0
330	*Contracaecum* spp.	Viscera	24
	*Posthodiplostomum* spp.	Liver	35
	*Leptorhynchoides* spp.	Stomach	1
		Pyloric Caeca	0
		Intestine	0
	*Neoechinorhynchus* spp.	Stomach	0
		Pyloric Caeca	0
		Intestine	9
	*Crepidostomum* spp.	Stomach	0
		Pyloric Caeca	9
		Intestine	12
	*Spinitectus* spp.	Stomach	0
		Pyloric Caeca	0
		Intestine	2
226	*Contracaecum* spp.	Viscera	2
	*Posthodiplostomum* spp.	Liver	22
	*Leptorhynchoides* spp.	Stomach	0
		Pyloric Caeca	0
		Intestine	0
	*Neoechinorhynchus* spp.	Stomach	0
		Pyloric Caeca	0
		Intestine	7
	*Crepidostomum* spp.	Stomach	0
		Pyloric Caeca	2
		Intestine	0
	*Spinitectus* spp.	Stomach	0
		Pyloric Caeca	0
		intestine	3
591	*Contracaecum* spp.	Viscera	7
	*Posthodiplostomum* spp.	Liver	22
	*Leptorhynchoides* spp.	Stomach	3
		Pyloric Caeca	3
		Intestine	0
	*Neoechinorhynchus* spp.	Stomach	0
		Pyloric Caeca	0
		Intestine	15
	*Crepidostomum* spp.	Stomach	0
		Pyloric Caeca	11
		Intestine	0
	*Spinitectus* spp.	Stomach	36
		Pyloric Caeca	1
		Intestine	0
369	*Contracaecum* spp.	Viscera	17
	*Posthodiplostomum* spp.	Liver	38
	*Leptorhynchoides* spp.	Stomach	1
		Pyloric Caeca	5
		Intestine	4
	*Neoechinorhynchus* spp.	Stomach	5
		Pyloric Caeca	5
		Intestine	12
	*Crepidostomum* spp.	Stomach	2
		Pyloric Caeca	26
		Intestine	0
	*Spinitectus* spp.	Stomach	2
		Pyloric Caeca	0
		intestine	4
158	*Contracaecum* spp.	Viscera	24
	*Posthodiplostomum* spp.	Liver	218
	*Leptorhynchoides* spp.	Stomach	0
		Pyloric Caeca	3
		Intestine	7
	*Neoechinorhynchus* spp.	Stomach	0
		Pyloric Caeca	0
		Intestine	4
	*Crepidostomum* spp.	Stomach	0
		Pyloric Caeca	22
		Intestine	4
	*Spinitectus* spp.	Stomach	0
		Pyloric Caeca	0
		intestine	0
400	*Contracaecum* spp.	Viscera	24
	*Posthodiplostomum* spp.	Liver	453
	*Leptorhynchoides* spp.	Stomach	1
		Pyloric Caeca	0
		Intestine	0
	*Neoechinorhynchus* spp.	Stomach	0
		Pyloric Caeca	0
		Intestine	4
	*Crepidostomum* spp.	Stomach	49
		Pyloric Caeca	290
		Intestine	3
	*Spinitectus* spp.	Stomach	7
		Pyloric Caeca	0
		intestine	10

*Note*: The fish are listed by their identifying number related to other studies. The number of parasite worms of each genus found in at each infection site is given including zeros where organs were searched but no parasitic worms were found.

**TABLE A4:** %C, %N, %P, C:N, C:P, and N:P compared across parasite genera collected from the digestive tract of 30 largemouth bass.

Parasites compared	C	N	P	C:N	C:P	N:P
*Contracaecum* spp. × *Leptorhynchoides* spp.	Diff = −4.92	**Diff = −5.20**	Diff = −0.24	**Diff = 2.84**	Diff = 107	Diff = −9.32
	*p* = .10	***p* = <.001**	*p* = .10	***p* = <.001**	*p* = .34	*p* = .84
*Contracaecum* spp. × *Neoechinorhynchus* spp.	Diff = −4.17	**Diff = −5.78**	Diff = −0.02	**Diff = 3.14**	Diff = 36.4	Diff = −22.4
	*p* = .16	***p* = <.001**	*p* = .99	***p* = <.0001**	*p* = .93	*p* = .16
*Contracaecum* spp. × *Posthodiplostomum* spp.	**Diff = −15.25**	**Diff = −1.97**	Diff = −0.20	Diff = −0.38	Diff = 82.3	Diff = 13.1
	***p* = <.0001**	***p* = .0009**	*p* = .22	*p* = .74	*p* = .40	*p* = .49
*Leptorhynchoides* spp. × *Neoechinorhynchus* spp.	Diff = 0.74	Diff = −0.58	Diff = 0.22	Diff = 0.30	Diff = −70.8	Diff = −13.1
	*p* = .99	*p* = .78	*p* = .16	*p* = .92	*p* = .72	*p* = .69
*Leptorhynchoides* spp. × *Posthodiplostomum* spp.	**Diff = −10.33**	**Diff = 3.23**	Diff = 0.04	**Diff = −3.22**	Diff = −24.9	Diff = 22.4
	***p* = .0008**	***p* = .0001**	*p* = .98	***p* = <.0001**	*p* = .99	*p* = .33
*Neoechinorhynchus* spp. × *Posthodiplostomum* spp.	**Diff = −11.07**	**Diff = 3.81**	Diff = −0.18	**Diff = −3.51**	Diff = 45.9	**Diff = 35.5**
	***p* = .0001**	***p* = .0001**	*p* = .50	***p* = .0001**	*p* = .91	***p* = .03**

*Note*: Diff is the difference between the means in Tukey post hoc, and *p* is the significance of the difference. Significant differences are indicated in bold.

**TABLE A5:** 5% confidence intervals for the nutrient mismatch (%C, %N, %P, C:N, C:P, and N:P) between parasite genera and the host tissues they were found in.

Parasite	%C	%N	%P	C:N	C:P	N:P
*Contracaecum* spp.	**2.5% = −11.89**	**2.5% = −5.93**	2.5% = −0.21	**2.5% = 0.09**	2.5% = −0.21	2.5% = −0.44
	**97.5% = −7.45**	**97.5% = −2.39**	97.5% = 0.11	**97.5% = 0.51**	97.5% = 0.29	97.5% = 0.06
*Leptorhynchoides* spp.	2.5% = −1.52	**2.5% = 4.08**	**2.5% = 0.13**	**2.5% = −0.89**	**2.5% = −1.22**	2.5% = −0.63
	97.5% = 6.71	**97.5% = 8.05**	**97.5% = 0.56**	**97.5% = −0.36**	**97.5% = −0.38**	97.5% = 0.23
*Neoechinorhynchus* spp.	2.5% = −3.46	**2.5% = 5.51**	2.5% = 0.04	**2.5% = −1.05**	**2.5% = −0.85**	2.5% = −0.14
	97.5% = 4.48	**97.5% = 9.31**	97.5% = 0.39	**97.5% = −0.55**	**97.5% = −0.02**	97.5% = 0.71
*Posthodiplostomum* spp.	**2.5%= 10.33**	2.5% = −0.55	**2.5% = 0.05**	2.5% = −0.09	2.5% = −0.73	**2.5% = −0.87**
	**97.5% = 18.27**	97.5% = 2.70	**97.5% = 0.51**	97.5% = 0.32	97.5% = 0.05	**97.5% = −0.06**

*Note*: Parasites came from the digestive tracts of 26 largemouth bass collected from the Mississippi river. The numbers in bold denote 97.5% confidence intervals that do not overlap zero.

**TABLE A6:** Emmeans pairwise comparison of %C of *Leptorhynchoides* spp*.* worms found across three different organs in 7 largemouth bass hosts. In this model fish ID was treated as a random effect.

	Estimate	SE	df	*t* ratio	*p*‐value
Intestine × Pyloric Caeca	−10.83	2.51	3.68	−4.32	.03
Intestine × Stomach	−10.02	3.27	5.50	−3.07	.05
Pyloric Caeca × Stomach	0.80	2.51	3.68	0.32	.95

**TABLE A7:** Model selection for the abundance of parasites within each genus and organ (Dependent variable = parasite genera), size of the infracommunity of each organ (Dependent variable = infra size of organ) as a function of each stoichiometric parameter of host organs, collection site, or a null model.

Dependent variable	Organ	Predictors	*K*	ΔAIC_C_	*W*	LL
*Neoechinorhynchus* spp.	Intestine	**Null**	**24**	**0.00**	**0.83**	**−18.49**
		Null + Pool	4	5.16	0.06	−18.16
	Pyloric Caeca	**Null**	**2**	**0.00**	**1**	**−5.26**
*Leptorhynchoides* spp.	Intestine	**Null**	**2**	**0.00**	**0.90**	**−4.95**
		Null + Pool	4	5.72	0.05	−2.67
	Pyloric Ceaca	**Null**	**2**	**0.00**	**0.84**	**−17.05**
		Null + Pool	4	3.94	0.12	−15.12
*Crepidostomum* spp.	Pyloric Caeca	**Null**	**2**	**0.00**	**0.39**	**−18.84**
		Null + Pool	4	0.24	0.35	−15.60
		C:N + Pool	5	4.14	0.05	−15.36
		%N + Pool	5	4.28	0.05	−15.44
		N:P + Pool	5	4.41	0.04	−15.50
		C:P + Pool	5	4.46	0.04	−15.23
		%C + Pool	5	4.52	0.04	−15.56
		%P + Pool	5	4.59	0.04	−15.59
*Posthodiplostomum* spp.	Liver	**Null**	**2**	**0.00**	**0.75**	**−30.31**
		Null + Pool	4	3.80	0.11	−29.30
		%C + Pool	5	5.88	0.04	−28.59
*Contracaecum* spp.	Viscera	**Null**	**2**	**0.00**	**0.77**	**−35.40**
		Null + Pool	4	5.14	0.06	−35.16
		%C + Pool	5	5.89	0.04	−33.89
Stomach		**Null**	**2**	**0.00**	**0.89**	**−14.51**
		Null + Pool	4	5.15	0.07	−13.73
Intestine		**Null**	**2**	**0.00**	**0.77**	**−28.05**
		Null + Pool	4	4.51	0.08	−27.64
Pyloric Caeca		**N:P + Pool**	**5**	**0.00**	**0.52**	**−26.61**
		Null + Pool	4	2.24	0.17	−26.03
		%P + Pool	5	3.94	0.07	−25.23
		Null	2	4.04	0.07	−29.74
		%N + Pool	5	4.65	0.05	−25.59
		C:P + Pool	5	4.80	0.05	−25.66
		C:N + Pool	5	4.97	0.04	−25.74
		%C + Pool	5	5.49	0.03	−26.00

*Note*: All models with the stoichiometry of host organs include collection site. Number of parameters (*K*), change in AICC compared to the best‐ranked model (ΔAIC_C_), Akaike model weights (*W*), and log likelihood estimate (LL) for top models (ΔAIC_C_ < 2) are shown in bold. Only models with a ΔAIC_C_ < 6 are included in this table.

**TABLE A8:** Model selection for the abundance of parasites within each genus (Dependent variable = parasite genera) as a function of the mismatch between parasite and host organ, collection site, or a null model.

Dependent Variable	Predictor	*K*	ΔAIC_C_	*W*	LL
*Contracaecum* spp.	**Null**	**2**	**0.00**	**0.86**	**−32.6**
	Null + Pool	4	5.35	0.06	−32.3
*Posthodiplostomum* spp.	**Null**	**2**	**0.00**	**0.54**	**−12.3**
	**Mismatch of %C**	**4**	**1.29**	**0.29**	**−7.83**
	Null + Pool	3	2.87	0.13	−11.6
*Neoechinorhynchus* spp.	**Null**	**2**	**0.00**	**0.97**	**−3.56**
*Leptorhynchoides* spp.	**Null**	**2**	**0.0**	**1**	**−2.91**

*Note*: Number of parameters (*K*), change in AICC compared to the best‐ranked model (ΔAIC_C_), Akaike model weights (*W*), and log likelihood estimate (LL) for top models (ΔAIC_C_ < 2) are shown in bold. Only models with a ΔAIC_C_ < 6 are included in this table.

## References

[ece311122-bib-0001] Aalto, S. L. , Decaestecker, E. , & Pulkkinen, K. (2015). A three‐way perspective of stoichiometric changes on host–parasite interactions. Trends in Parasitology, 31(7), 333–340. 25978937 10.1016/j.pt.2015.04.005

[ece311122-bib-0002] Altman, I. , & Byers, J. E. (2014). Large‐scale spatial variation in parasite communities influenced by anthropogenic factors. Ecology, 95(7), 1876–1887. 25163120 10.1890/13-0509.1

[ece311122-bib-0003] APHA . (1992). Standard methods for the examination of water and wastewater (18th ed.). American Public Health Association.

[ece311122-bib-0004] Bangham, R. V. (1939). Parasites of Centrarchidae from southern Florida. Transactions of the American Fisheries Society, 68(1), 263–268.

[ece311122-bib-0005] Bernot, R. J. , & Poulin, R. (2018). Ecological stoichiometry for parasitologists. Trends in Parasitology, 34(11), 928–933. 30104137 10.1016/j.pt.2018.07.008

[ece311122-bib-0006] Buddington, R. K. , & Diamond, J. M. (1986). Aristotle revisited: The function of pyloric caeca in fish. Proceedings of the National Academy of Sciences, 83(20), 8012–8014. 10.1073/pnas.83.20.8012 PMC386855 3464017

[ece311122-bib-0007] Chappell, L. H. (1980). Physiology of parasites. Wiley.

[ece311122-bib-0008] Choquette, L. P. E. (1954). A note on the intermediate hosts of the trematode, Crepidostomum cooperi Hopkins, 1931, parasitic in speckled trout (*Salvelinus fontinalis* (Mitchill)) in some lakes and rivers of the Quebec Laurentide Park. Canadian Journal of Zoology, 32(6), 375–377.

[ece311122-bib-0009] Costa, A. P. L. , Takemoto, R. M. , & Vitule, J. R. S. (2018). Metazoan parasites of *Micropterus salmoides* (Lacépède 1802) (Perciformes, Centrarchidae): A review with evidences of spillover and spillback. Parasitology Research, 117(6), 1671–1681. 29704120 10.1007/s00436-018-5876-9

[ece311122-bib-0010] de Buron, I. , & Nickol, B. B. (1994). Histopathological effects of the acanthocephalan *Leptorhynchoides thecatus* in the ceca of the green sunfish, *Lepomis cyanellus* . Transactions of the American Microscopical Society, 113, 161–168.

[ece311122-bib-0011] Dobson, A. , Lafferty, K. D. , Kuris, A. M. , Hechinger, R. F. , & Jetz, W. (2008). Homage to Linnaeus: How many parasites? How many hosts? Proceedings of the National Academy of Sciences, 105, 11482–11489. 10.1073/pnas.0803232105 PMC2556407 18695218

[ece311122-bib-0012] Elser, J. J. , Dobberfuhl, D. R. , Mackay, N. A. , & Schampel, J. H. (1996b). Organism size, life history, and N: P stoichiometry toward a unified view of cellular and ecosystem processes. Bioscience, 46, 674–684.

[ece311122-bib-0013] Elser, J. J. , Dobberfuhl, D. R. , MacKay, N. A. , & Schampel, J. H. (1996a). Organism size, life history, and N:P stoichiometry: Toward a unified view of cellular and ecosystem processes. Bioscience, 46(9), 674–684. 10.2307/1312897

[ece311122-bib-0014] Færøvig, P. J. , & Hessen, D. O. (2003). Allocation strategies in crustacean stoichiometry: The potential role of phosphorus in the limitation of reproduction. Freshwater Biology, 48, 1782–1792.

[ece311122-bib-0015] Frenken, T. , Paseka, R. , González, A. L. , Asik, L. , Seabloom, E. W. , White, L. A. , Borer, E. T. , Strauss, A. T. , Peace, A. , & Van de Waal, D. B. (2021). Changing elemental cycles, stoichiometric mismatches, and consequences for pathogens of primary producers. Oikos, 130(7), 1046–1055.

[ece311122-bib-0016] Fried, B. , & Graczyk, T. K. (Eds.). (1997). Advances in trematode biology. CRC Press.

[ece311122-bib-0017] Frost, P. C. , Ebert, D. , & Smith, V. H. (2008). Responses of a bacterial pathogen to phosphorus limitation of its aquatic invertebrate host. Ecology, 89(2), 313–318. 18409420 10.1890/07-0389.1

[ece311122-bib-0018] Hendrixson, H. A. , Sterner, R. W. , & Kay, A. D. (2007). Elemental stoichiometry of freshwater fishes in relation to phylogeny, allometry and ecology. Journal of Fish Biology, 70(1), 121–140.

[ece311122-bib-0019] Hoffman, G. L. (1958). Experimental studies on the cercaria and metacercaria of a strigeoid trematode, Posthodiplostomum Minimum. Experimental Parasitology, 7(1), 23–50. 13501233 10.1016/0014-4894(58)90004-3

[ece311122-bib-0020] Hoffman, G. L. , & Williams, E. H. (1999). Parasites of north American freshwater fishes (2nd ed.). Cornell University Press.

[ece311122-bib-0021] Howick, G. L. , & OˈBrien, W. J. (1983). Piscivorous feeding behavior of largemouth bass: An experimental analysis. Transactions of the American Fisheries Society, 112(4), 508–516.

[ece311122-bib-0022] Huizinga, H. W. (1967). The life cycle of Contracaecum multipapillatum (von Drasche, 1882) Lucker, 1941 (Nematoda: Heterochelidae). The Journal of Parasitology, 53, 368–375. 6067110

[ece311122-bib-0023] Hunter, G. W. , & Hunter, W. S. (1940). Studies on the development of the metacercaria and the nature of the cyst of Posthodiplostomum minimum (MacCallum 1921) (Trematoda; Strigeata). Transactions of the American Microscopical Society, 59(1), 52–63. 10.2307/3222816

[ece311122-bib-0024] Johnson, P. T. , Chase, J. M. , Dosch, K. L. , Hartson, R. B. , Gross, J. A. , Larson, D. J. , Sutherland, D. R. , Carpenter, S. R. , & Carpenter, S. R. (2007). Aquatic eutrophication promotes pathogenic infection in amphibians. Proceedings of the National Academy of Sciences, 104(40), 15781–15786. 10.1073/pnas.0707763104 PMC2000446 17893332

[ece311122-bib-0025] Johnson, P. T. , Townsend, A. R. , Cleveland, C. C. , Glibert, P. M. , Howarth, R. W. , McKenzie, V. J. , Rejmankova, E. , & Ward, M. H. (2010). Linking environmental nutrient enrichment and disease emergence in humans and wildlife. Ecological Applications, 20(1), 16–29. 20349828 10.1890/08-0633.1 PMC2848386

[ece311122-bib-0026] Justić, D. , Rabalais, N. N. , & Turner, R. E. (2002). Modeling the impacts of decadal changes in riverine nutrient fluxes on coastal eutrophication near the Mississippi River Delta. Ecological Modelling, 152(1), 33–46.

[ece311122-bib-0027] Klein, W. D. , Olsen, O. W. , & Bowden, D. C. (1969). Effects of intestinal fluke, Crepidostomum farionis, on rainbow trout, Salmo gairdnerii. Transactions of the American Fisheries Society, 98(1), 1–6.

[ece311122-bib-0028] Knox, D. P. (2007). Proteinase inhibitors and helminth parasite infection. Parasite Immunology, 29(2), 57–71. 17241394 10.1111/j.1365-3024.2006.00913.x

[ece311122-bib-0029] Kong, H. , Edberg, D. D. , Korte, J. J. , Salo, W. L. , Wright, P. A. , & Anderson, P. M. (1998). Nitrogen excretion and expression of carbamoyl‐phosphate synthetase III activity and mRNA in extrahepatic tissues of largemouth bass (*Micropterus salmoides*). Archives of Biochemistry and Biophysics, 350(2), 157–168. 9473289 10.1006/abbi.1997.0522

[ece311122-bib-0030] Leadabrand, C. , & Nickol, B. (1993). Establishment, survival, site selection and development of *Leptorhynchoides thecatus* in largemouth bass, Micropterus Salmoides. Parasitology, 106(5), 495–501. 10.1017/S003118200007679417/S0031182000076794 8341586

[ece311122-bib-0031] Lenth, R. (2022). emmeans: Estimated marginal means, aka least‐squares Means. R package version 1.8.2. https://CRAN.R‐project.org/package=emmeans

[ece311122-bib-0032] Lowenberger, C. A. , & Rau, M. E. (1993). Plagiorchis elegans: Requirements for metacercarial development to infectivity, and conditions required for excystment. Journal of Helmithological Society of Washington, 60, 67–71.

[ece311122-bib-0033] McKenzie, V. J. , & Townsend, A. R. (2007). Parasitic and infectious disease responses to changing global nutrient cycles. EcoHealth, 4(4), 384–396.

[ece311122-bib-0034] Mehlhorn, H. (Ed.). (2001). Acanthocephala. In Encyclopedic reference of parasitology. Springer. 10.1007/3-540-29834-7_8

[ece311122-bib-0035] Miller, J. H. (1953). Studies on the life history of Posthodiplostomum minimum (Mac Callum, 1921). New York University. 13184371

[ece311122-bib-0036] Mischler, J. , Johnson, P. T. , McKenzie, V. J. , & Townsend, A. R. (2016). Parasite infection alters nitrogen cycling at the ecosystem scale. Journal of Animal Ecology, 85(3), 817–828. 26919319 10.1111/1365-2656.12505

[ece311122-bib-0037] Nakagawa, S. , & Schielzeth, H. (2013). A general and simple method for obtaining *R* ^ *2* ^ from generalized linear mixed‐effects models. Methods in Ecology and Evolution, 4, 133–142.

[ece311122-bib-0038] Narr, C. F. , Ebert, D. , Bastille‐Rousseau, G. , & Frost, P. C. (2019). Nutrient availability affects the prevalence of a microsporidian parasite. Journal of Animal Ecology, 88(4), 579–590. 30636044 10.1111/1365-2656.12945

[ece311122-bib-0039] Narr, C. F. , & Frost, P. C. (2016). Exploited and excreting: Parasite type affects host nutrient recycling. Ecology, 97(8), 2012–2020. 27859196 10.1002/ecy.1437

[ece311122-bib-0040] Narr, C. F. , & Krist, A. C. (2015). Host diet alters trematode replication and elemental composition. Freshwater Science, 34(1), 81–91.

[ece311122-bib-0041] Paseka, R. E. , & Grunberg, R. L. (2019). Allometric and trait‐based patterns in parasite stoichiometry. Oikos, 128(1), 102–112.

[ece311122-bib-0042] Persson, J. , Fink, P. , Goto, A. , Hood, J. M. , Jonas, J. , & Kato, S. (2010). To be or not to be what you eat: Regulation of stoichiometric homeostasis among autotrophs and heterotrophs. Oikos, 119(5), 741–751.

[ece311122-bib-0043] Poulin, R. (2011). Evolutionary ecology of parasites. In Evolutionary ecology of parasites. Princeton University Press.

[ece311122-bib-0044] Ratcliff, E. N. , Gittinger, E. J. , O'Hara, T. M. , & Ickes, B. S. (2014). Long term resource monitoring program procedures: Fish monitoring. US Army Corps of Engineers, Upper Mississippi River restoration–Environmental management program, report LTRMP‐2014‐P001. USGS.

[ece311122-bib-0045] Raubenheimer, D. , & Simpson, S. J. (2004). Organismal stoichiometry: Quantifying non‐independence among food components. Ecology, 85(5), 1203–1216.

[ece311122-bib-0046] Reiners, W. A. (1986). Complementary models for ecosystems. The American Naturalist, 127(1), 59–73.

[ece311122-bib-0047] Ricker, W. E. (1975). Computation and interpretation of biological statistics of fish populations. Bulletin–Fisheries Research Board of Canada, 191, 1–382.

[ece311122-bib-0048] Sanders, A. J. , & Taylor, B. W. (2018). Using ecological stoichiometry to understand and predict infectious diseases. Oikos, 127(10), 1399–1409.

[ece311122-bib-0049] Sarbahi, D. S. (1951). Studies of the digestive tracts and the digestive enzymes of the goldfish, *Carassius auratus* (linnaeus) and the largemouth black bass, *Micropterus salmoides* (lacépède). The Biological Bulletin, 100(3), 244–257. 10.2307/1538534 14838933

[ece311122-bib-0050] Smith, V. H. , Holt, R. D. , Smith, M. S. , Niu, Y. , & Barfield, M. (2015). Resources, mortality, and disease ecology: Importance of positive feedbacks between host growth rate and pathogen dynamics. Israel Journal of Ecology & Evolution, 61(1), 37–49. 27642269 10.1080/15659801.2015.1035508 PMC5026129

[ece311122-bib-0051] Sterner, R. W. , & Elser, J. J. (2002). Ecological stoichiometry: The biology of elements from molecules to the biosphere. Princeton University Press.

[ece311122-bib-0052] Tilman, D. (1982). Resource competition and community structure. Princeton University Press. 7162524

[ece311122-bib-0053] Turk, V. , Stoka, V. , & Turk, D. (2008). Cystatins: Biochemical and structural properties, and medical relevance. Frontiers in Bioscience‐Landmark, 13(14), 5406–5420. 10.2741/3089 18508595

[ece311122-bib-0054] Van Cleave, H. J. (1920). Notes on the life cycle of two species of Acanthocephala from freshwater fishes. The Journal of Parasitology, 6(4), 167–172.

[ece311122-bib-0055] Venard, C. E. , & Warfel, J. H. (1953). Some effects of two species of Acanthocephala on the alimentary canal of the largemouth bass. The Journal of Parasitology, 39(2), 187–190. 13045187

[ece311122-bib-0056] Von Brand, T. (1952). Chemical physiology of endoparasitic animals. Academic Press.

[ece311122-bib-0057] Vrede, T. , Drakare, S. , Eklöv, P. , Hein, A. , Liess, A. , Olsson, J. , Persson, J. , Quevedo, M. , Stabo, H. R. , & Svanbäck, R. (2011). Ecological stoichiometry of Eurasian perch–intraspecific variation due to size, habitat and diet. Oikos, 120(6), 886–896.

[ece311122-bib-0058] Ward, H. L. (1940). Studies on the life history of Neoechinorhynchus cylindratus (Van Cleave, 1913) (Acanthocephala). Transactions of the American Microscopical Society, 59(3), 327–347.

[ece311122-bib-0059] Zuzarte‐Luís, V. , & Mota, M. M. (2018). Parasite sensing of host nutrients and environmental cues. Cell Host & Microbe, 23(6), 749–758. 29902440 10.1016/j.chom.2018.05.018

